# Cooperativity of Electron Transfer Coupled Spin Transitions in a Tetranuclear Fe/Co Prussian Blue Analogue Revealed by Ultrafast Spectroscopy

**DOI:** 10.1002/anie.202505813

**Published:** 2025-05-09

**Authors:** Jan‐Hendrik Borter, Simindokht Gol Kar, Sayan Kangsa Banik, Serhiy Demeshko, Rainer Oswald, Martí Gimferrer, Ricardo A. Mata, Dirk Schwarzer, Franc Meyer

**Affiliations:** ^1^ Department of Dynamics at Surfaces Max‐Planck‐Institute for Multidisciplinary Sciences Am Fassberg 11 37077 Göttingen Germany; ^2^ Institute for Inorganic Chemistry Georg‐August‐Universität Göttingen Tammannstrasse 4 37077 Göttingen Germany; ^3^ Institute for Physical Chemistry Georg‐August‐Universität Göttingen Tammannstrasse 6 37077 Göttingen Germany

**Keywords:** Cooperativity, Electron transfer coupled spin transition, Kinetic modelling, Prussian blue analogues, Ultrafast spectroscopy

## Abstract

Cooperative intermolecular interactions, usually observed in solid state, can confer useful properties to stimuli–responsive spin transition materials. Here, we demonstrate for the first time intramolecular cooperativity between the two Fe–Co subunits of a molecular cyanido‐bridged square Fe_2_Co_2_ Prussian blue analogue (PBA) in solution, which upon single photon excitation sequentially undergo electron transfer coupled spin transition (ETCST) from a diamagnetic low‐spin (LS) to a paramagnetic high‐spin (HS) state. Ultrafast UV–vis and IR pump‐probe spectroscopies show that irradiation into the IVCT band of the LS state induces electron transfer within one Fe–Co subunit followed by fast (360 fs) SCO to an intermediate HS/LS species and a further ETCST event in the other Fe–Co subunit then occurs on a ns timescale. Kinetic analysis reveals that this cooperative switching of the two Fe–Co subunits is caused by two coupled equilibria favouring the second ETCST step, and the free energy landscape for the square Fe_2_Co_2_ system is determined experimentally.

## Graphical abstract

Ultrafast pump‐probe spectroscopies show that irradiation of a square Fe/Co Prussian blue analogue induces electron transfer within one Fe–Co subunit followed by fast spin crossover, and coupled equilibria between all states trigger a second electron transfer coupled to spin crossover in the other Fe–Co subunit on a ns timescale. This represents a unique case of cooperative two‐step switching on a molecular level.

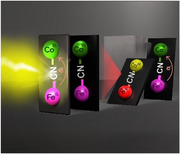

## Introduction

**Figure 1:**
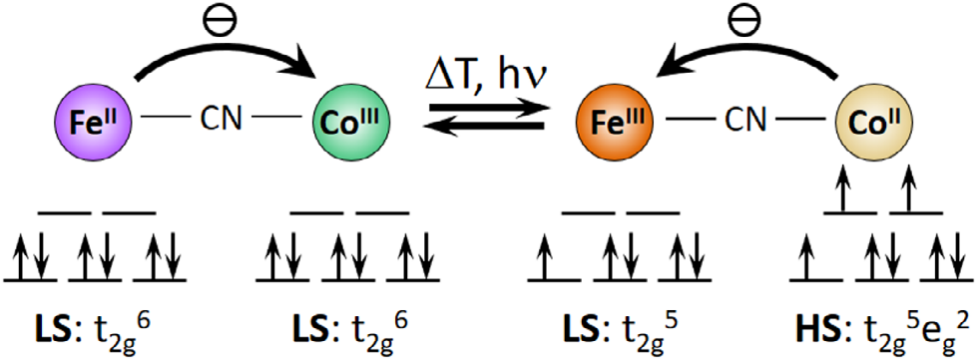
Interconversion between the diamagnetic LT (left) and paramagnetic HT (right) electronic configurations of a Fe/Co PBA entity caused by ETCST (LS = low spin, HS = high‐spin).

Prussian blue analogues (PBAs) are a family of cyano‐bridged bimetallic coordination compounds that are well known for their switchable electronic states.^[^ ^1^, ^2^, ^3^ ^]^ PBAs exhibit a wide range of magnetic and optical properties, making them attractive for various applications such as molecular‐level information processing,^[^ ^4^ ^]^ optical displays^[^ ^5^ ^]^ and sensor technology.^[^ ^6^ ^]^ In Fe/Co PBAs, external stimuli such as temperature changes or light absorption can cause reversible intramolecular Fe/Co electron transfer (ET) coupled with spin‐crossover (SCO) on the Co centre, i.e., a diamagnetic cyanido‐bridged Fe^II^_LS_Co^III^_LS_ unit with Fe(t_2g_^6^e_g_^0^)/Co(t_2g_^6^e_g_^0^) configuration—the low temperature species (LT)—transforms into the paramagnetic Fe^III^_LS_Co^II^_HS_ state with Fe(t_2g_^5^e_g_^0^)/Co(t_2g_^5^e_g_^2^) configuration—the high temperature species (HT) (see Figure 1). This phenomenon is called electron transfer coupled spin transition (ETCST) or sometimes also charge transfer‐induced spin transition (CTIST).^[^ ^2^ ^]^ Oligonuclear Co/Fe PBAs that feature thermally induced multistep ETCST signatures have recently been reported.^[^ ^7^, ^8^, ^9^ ^]^

**Figure 2:**
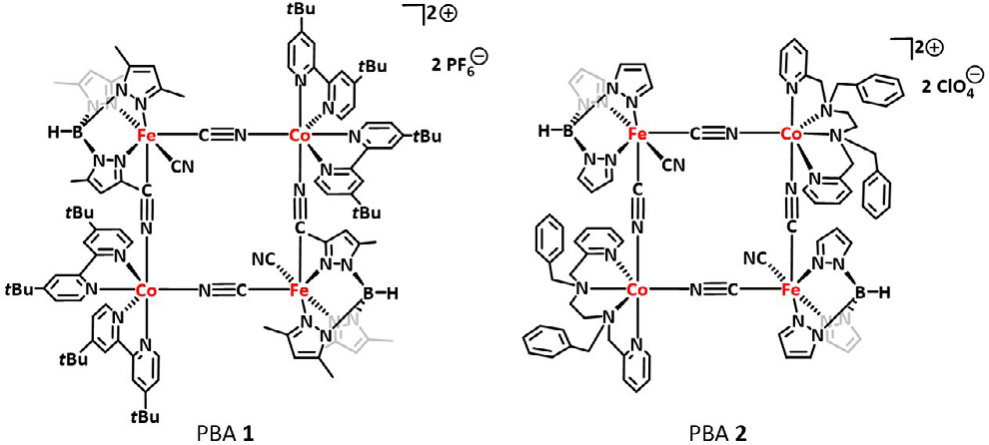
Structures of PBA **1** ^[^ ^7^ ^]^ previously studied by us via fs mid‐IR and UV–vis pump‐probe spectroscopy^[^ ^12^ ^]^ and PBA **2** ^[^ ^13^ ^]^ investigated in this work.

In recent years, the light‐induced dynamics in PBAs have been investigated by applying ultrafast spectroscopy in order to decipher the sequence of reaction steps leading to ETCST.^[^ ^10^ ^]^ Using X‐ray and optical absorption spectroscopy, Cammarata et al.^[^ ^11^ ^]^ concluded that in a prototypical PBA system consisting of nanocrystals of the cyano‐bridged Fe^II^Co^III^ compound Cs_0.7_Co[Fe(CN)_6_]_0.9_, light absorption at 583 nm induces first a SCO on the Co centre within ∼50 fs, followed by a Fe → Co electron transfer on a 200 fs timescale. By contrast, for a molecular square‐type Fe^II^_2_Co^III^_2_ Prussian blue analogue^[^ ^7^ ^]^ (PBA **1**, see Figure 2) in solution, we observed a different order, where after photoexcitation at 775 or 800 nm into the intervalence charge transfer (IVCT) band direct electron transfer within one Fe–Co edge of the square complex occurs, which after 400 fs undergoes SCO to form a long‐lived Fe^III^_LS_Co^II^_HS_ entity.^[^ ^12^ ^]^

Another important aspect of switching valence and spin states in PBAs concerns the role of cooperativity enabling several Fe/Co entities to undergo ETCST by absorbing a single photon. This effect was previously observed for crystalline PBA samples, which by pulsed high intensity irradiation showed phase transitions from the low temperature Fe^II^_LS_Co^III^_LS_ phase to the high temperature Fe^III^_LS_Co^II^_HS_ phase.^[^ ^14^, ^15^ ^]^Similar cooperative effects have been observed in molecular crystals of mononuclear SCO compounds, such as Fe(phen)_2_(NCS)_2_, where local ultrafast photoswitching from low‐spin (LS) to high‐spin (HS) states causes additional conversion of further molecules in the crystal lattice on ns to µs timescales due to elastic and thermal effects.^[^ ^16^, ^17^ ^]^ In case of an oligonuclear grid‐type Fe_4_ complex with four ligand‐bridged (strongly elastically coupled) Fe^II^ ions, time‐resolved crystallography revealed that these long‐range elastic and thermal steps are preceded by short‐range intramolecular distortions propagating over the entire metallogrid on a ps timescale due to the continuous rearrangement of the Fe_4_ molecule triggered by the photoinduced LS →HS switching at a single Fe^II^ site.^[^ ^18^ ^]^

In this work, we investigated the molecular square‐type PBA [Fe(Tp)(CN)_3_]_2_[Co{en(Bn)py}]_2_(ClO_4_)_2_, recently presented by Yadav et al.^[^ ^13^ ^]^ (PBA **2** in Figure 2; Tp = tris(pyrazolyl)borate; en(Bn)py = *N* ^1^,*N* ^2^‐dibenzyl‐*N* ^1^,*N* ^2^‐bis(pyridine‐2‐ylmethyl)ethane‐1,2‐diamine). Crystalline material of **2** was reported to exhibit a thermal ETCST well above room temperature with wide hysteresis window.^[^ ^13^ ^]^ We have now studied this complex in acetonitrile solution using fs mid‐IR and UV–vis pump‐probe spectroscopy to unravel the dynamics of individual molecules after light excitation. Our data confirm our previous results for PBA **1** that excitation into the IVCT band of the diamagnetic LS state of PBA **2** induces immediate electron transfer within one Fe–Co subunit followed by fast SCO to a [Fe^II^_LS_Co^III^_LS_Fe^III^_LS_Co^II^_HS_] species. Interestingly, our data clearly indicate a subsequent second ETCST step on a ns timescale involving the other Fe–Co subunit of PBA **2**. This is the first time that single‐photon‐induced cooperative ETCST, caused by coupling of the two Fe–Co units, has been demonstrated at the molecular level.

## Results

### Synthesis and Solid‐State Characterization

The tetranuclear Fe_2_Co_2_ PBA **2** was synthesised according to the reported procedure;^[^ ^13^ ^]^ details of the synthesis and characterization are presented in the Supporting Information. Single‐crystal X‐ray diffraction and SQUID measurements of the prepared material were consistent with the reported data. Additionally, a ^57^Fe Mössbauer spectrum was recorded to complete the characterization of solid PBA **2** and to confirm the purity of the material used. The Mössbauer spectrum at 80 K shows a single quadrupole doublet with an isomer shift of *δ* = 0.17 mms^−1^ and a quadrupole splitting of Δ*E*_Q_ = 0.47 mms^−1^ characteristic for the Fe^II^_LS_ state.

### UV–Vis and FTIR Spectra in Solution

**Figure 3:**
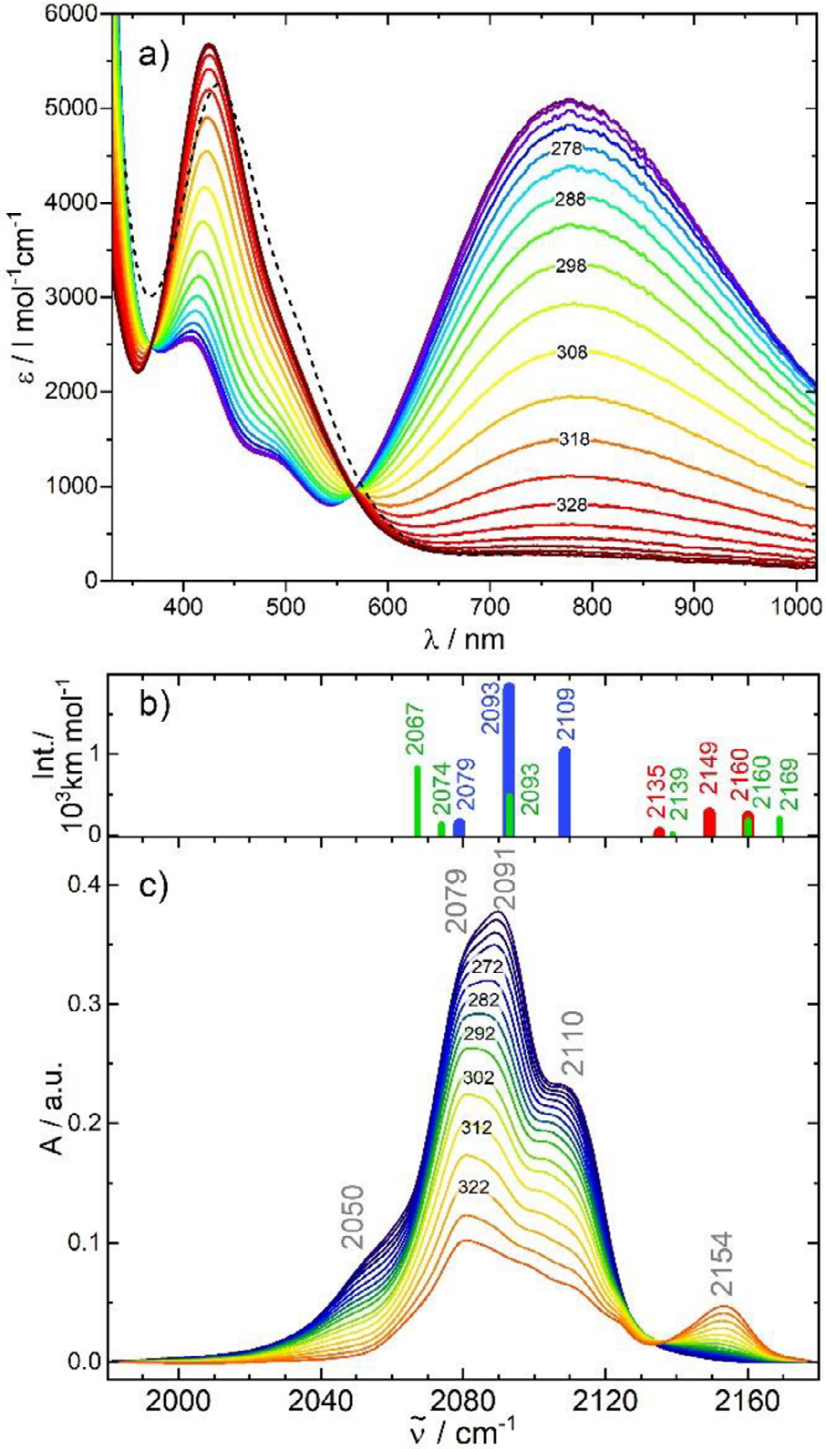
a) UV–vis and c) FTIR absorption spectra of PBA **2** in acetonitrile solution at indicated temperatures (in K; spectra were recorded at 253–348 K and 252–332 K, respectively, in steps of Δ*T* = 5 K). The dashed line in (a) corresponds to the UV–vis spectrum of the one‐electron transfer product ET_1_ ([Fe^II^_LS_Co^III^_LS_Fe^III^_LS_Co^II^_HS_]) derived from transient absorption spectroscopy, see text. Panel b) shows stick spectra for the LT (blue), HT (red), and ET_1_ (green) species computed by DFT.

The integrity of PBA **2** in acetonitrile solution was first verified by ESI(+) mass spectrometry (see Supporting Information) showing a main peak at *m*/*z* = 828.2 with isotopic distribution pattern characteristic of the doubly charged cation of PBA **2**. Thermal stability was further tested by recording temperature dependent UV–vis and FTIR spectra in the range 250–350 K (Figures 3 and S5). Both demonstrate the transition from the LT [Fe^II^_LS_Co^III^_LS_]_2_ to the HT [Fe^III^_LS_Co^II^_HS_]_2_ species. Under inert conditions at *T* < 330 K, the transformation is completely reversible and shows no hysteresis. For higher temperatures, we observe slow decomposition into smaller monometallic fragments (details are presented in Supporting Information).

The UV–vis spectrum of the low temperature species in Figure 3a exhibits a broad intense IVCT band centred at 770 nm and a narrower band of lower intensity at 410 nm with a shoulder at 490 nm (a Mulliken–Hush analysis of the IVCT absorption band is included in the Supporting Information). With increasing temperature, the LT IVCT band disappears and the spectrum of the HT species with a maximum at 420 nm emerges. The isosbestic point at 565 nm suggests a simultaneous thermally induced electron transfer in both Fe/Co subunits of the square molecule. However, the detailed analysis of our kinetic data presented below will unveil a stepwise electron transfer involving a one‐electron IVCT intermediate (ET_1_), albeit its equilibrium concentration appears to be below the detection limit. The experimental FTIR spectra, Figure 3c, showing the thermal ETCST by means of the CN stretching vibrations of PBA **2** are consistent with these results. The low temperature spectrum shows bands at *ν*_CN_ = 2050, 2080, 2090 and 2109 cm^−1^. Upon heating, the intensity of these transitions drops and a band at 2154 cm^−1^ appears, being a marker for the HT species. A similar signature was previously observed for PBA **1**.^[^ ^7^, ^12^ ^]^

**Figure 4:**
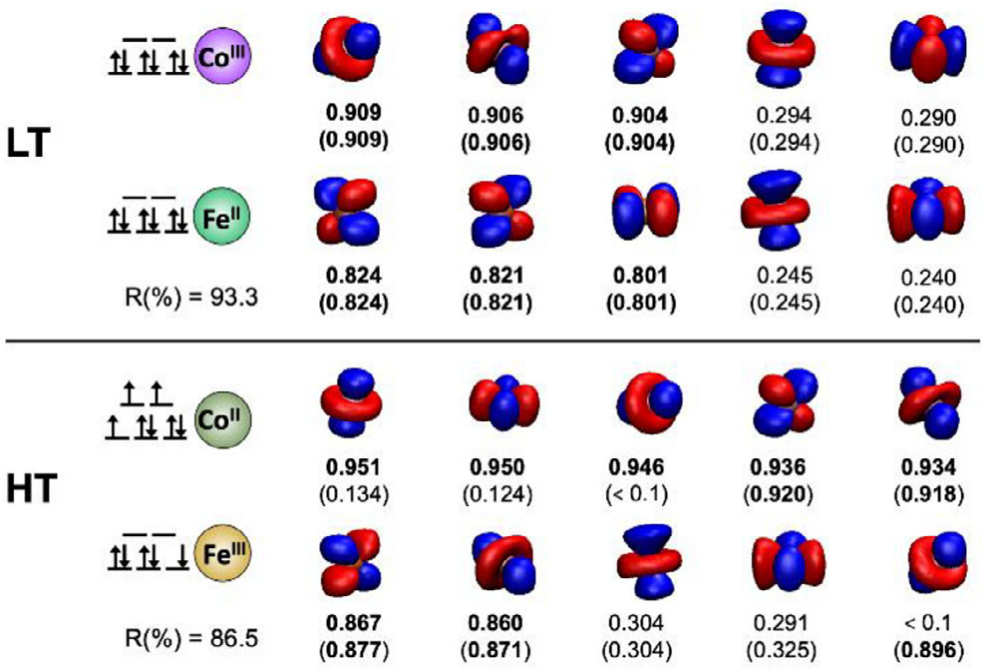
LC‐BP86/def2‐TZVP EFO analysis (metal d orbitals) of converged electronic states for the (LT) and high‐temperature (HT) regimes. Under each EFO, we include the occupation of α‐ and β‐orbitals (top and bottom in parenthesis, respectively). The LT calculations were carried out under a restricted Kohn–Sham formalism, the HT unrestricted.

We have carried out DFT calculations to assign the relevant bands of the FTIR spectra, considering different oxidation and spin states for the metal ions. These calculations are described in more detail later in the text, but the results appear consistent with our study of PBA **1**. The LT state consists of Fe^II^_LS_ and Co^III^_LS_ ions. With increasing temperature, an electron transfer occurs, leading to Fe^III^_LS_ and Co^II^_HS_ ions that are antiferromagnetically coupled. In order to confirm the electronic structure assignments, we calculated the effective oxidation states (EOS) from the effective fragment orbitals (EFOs)^[^ ^19^, ^20^, ^21^ ^]^ and their occupation values (see Figure 4) with the APOST‐3D program package.^[^ ^22^ ^]^ The oxidation and spin states are confirmed by the EFO occupations, with *R*(%) values above 80% in all cases. The latter present a measure of how close the actual electronic structure is to the ionic assignment.

In order to support the FTIR measurements, we calculated the vibrational spectra for the HT and LT states. The computed CN stretching band positions and intensities are derived from the double‐harmonic approximation, with their position scaled to match the band of the LT state at 2079 cm^−1^. The scaling factor (0.901) accounts for both anharmonicity effects and shortcomings of the electronic structure method. The band positions for the LT state provide a good match with the measurements (see Figure 3b). The experimental band positions at 2091 and 2110 cm^−1^ are accurately reproduced by the scaled harmonic values, 2093 and 2109 cm^−1^. The features for the HT regime are not fully captured, with only the absorption band at higher wavenumbers (around 2154 cm^−1^) reproduced. We argue that the experimental HT profile between 2050 and 2130 cm^−1^ might be mainly caused by persisting LT states (Figure 3a shows that even above 330 K, there is no complete conversion).

Based on the temperature dependent UV–vis spectra, the van't Hoff equation was utilised to calculate the reaction enthalpy and entropy for the LT ⇄ HT equilibrium of PBA **2**, resulting in Δ*H* = 74 ± 7 kJ·mol^−1^ and Δ*S* = 240 ± 15 J·mol^−1^K^−1^, respectively, with a transition temperature at *T*_1/2_ = 305 ± 2 K (for details see Supporting Information). These values are similar to those measured for PBA **1** in acetonitrile (Δ*H* = 79 kJ·mol^−1^, Δ*S* = 299 J·mol^−1^K^−1^).^[^ ^12^ ^]^ However, they differ markedly from those reported for crystalline samples of PBA **2**·4MeOH·2H_2_O (Δ*H* = 162 kJ·mol^−1^, Δ*S* = 468 J·mol^−1^K^−1^),^[^ ^13^ ^]^ likely reflecting substantial effects of the solvent reorganisation upon electron transfer.

### Femtosecond Pump‐Probe Spectroscopy

Using ultrafast UV–vis and mid‐IR absorption spectroscopy, we studied solutions of PBA **2** in acetonitrile after light excitation with a time resolution of 100 and 200 fs, respectively (for details see Supporting Information). Experiments were performed at 298 and 318 K, where the equilibrium mixture contains 33% and 77% of the HT form of the complex, respectively. However, by applying 800 nm pump pulses, it is possible to selectively excite and follow the dynamics of the LT species as the HT species does not absorb at wavelengths >650 nm. On the other hand, thermal instability of PBA **2** in acetonitrile at *T* > 330 K prohibited reliable investigation of the HT species because its selective excitation in the presence of the LT species is not possible (cf. Figure 3a).

**Figure 5:**
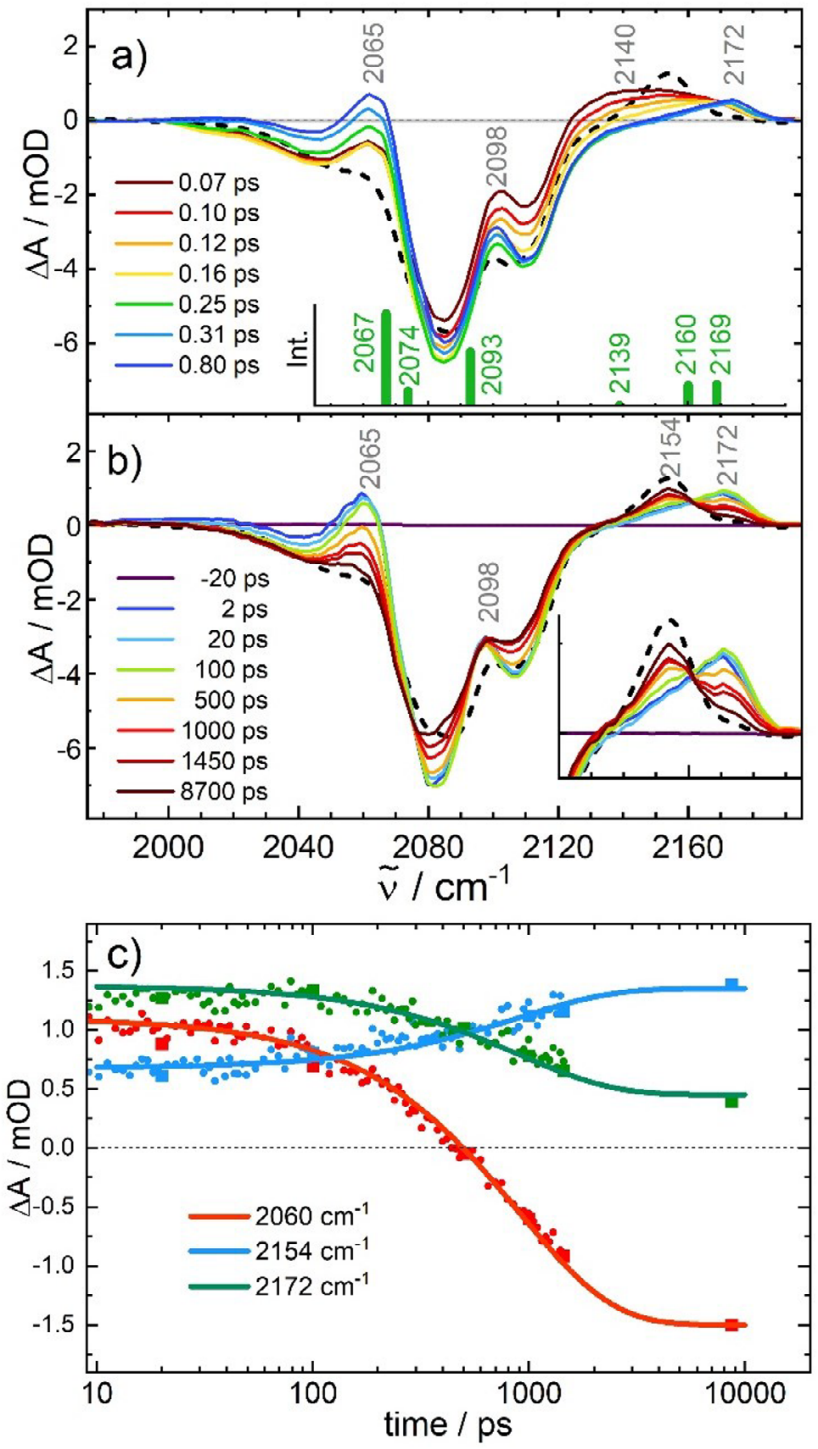
Transient difference spectra of the LT phase of PBA **2** in acetonitrile at 298 K in the region of the CN stretching vibrations following 800 nm excitation. Panel a) and b) show transients at short and long pump‐probe delays, respectively. The inset in (a) shows the calculated stick spectrum of ET_1_, and the inset in (b) enlarges the transformation of the 2172 into the 2154 cm^−1^ band. Black dashed lines represent a scaled difference spectrum, (**A**_332K_ − **A**_292K_), calculated from the FTIR spectra of Figure 3c. Panel c) shows time traces for specific IR bands with exponential fits yielding a global time constant of 900 ± 100 ps (note the logarithmic time axis).

Figure 5 shows absorbance changes, Δ*A* (in units of milli optical density, mOD), of the CN stretching bands after 800 nm excitation of the LT species of PBA **2** at 298 K. For reference, black dashed lines in Figure 5a,b represent the expected spectrum for an LT→HT transition involving ETCST in both Fe–Co subunits of the square complex (calculated from the difference *A*_332K_ − *A*_292K_ of the FTIR spectra at 332 and 292 K in Figure 3c). Although the spectrum of the parent molecule after excitation is instantaneously bleached, two features at 2065 and 2140 cm^−1^ arise, which undergo relaxation towards a longer‐lived product spectrum in a time range of a few hundred fs (Figure 5a). During this process, the broad feature at 2140 cm^−1^ blueshifts to a narrower band centred at 2172 cm^−1^, and concomitantly, the 2065 cm^−1^ peak gains intensity and develops a redshifted tail. A closer comparison of the IR transients with the bleached LT spectrum reveals an additional absorption band in the product spectrum at 2098 cm^−1^ (for detail see Figure S14). The marked differences in peak positions of the product spectrum to those of the HT species indicate that an absorbed pump photon induces electron transfer in only one Fe–Co unit of PBA **2** under formation of [Fe^II^Co^III^Fe^III^Co^II^]; we denote this one‐electron IVCT intermediate as ET_1_.

Electronic structure calculations were used to confirm the character of the ET_1_ state. The scaled harmonic frequencies for a [Fe^II^_LS_Co^III^_LS_Fe^III^_LS_Co^II^_HS_] configuration (see inset in Figure 5a) are in reasonable agreement with the IR pump‐probe measurements. The bands around 2065, 2098 and 2172 cm^−1^ are reproduced (2067, 2093 and 2160/2169 cm^−1^, respectively). The electronic state of the computed ET_1_ species was characterised by EFO analysis, confirming the attributed spin state distribution, with one Fe–Co unit in the same configuration as in the LT regime [Fe^II^_LS_Co^III^_LS_] and the other as in HT [Fe^III^_LS_Co^II^_HS_] (Figure 4, albeit the orbital shapes and occupations slightly differ).

Figure 5b illustrates events occurring on significantly longer timescales. First, the redshifted tail of the 2065 cm^−1^ band disappears with a time constant of 20 ± 5 ps, indicating that the [Fe^II^Co^III^Fe^III^Co^II^] intermediate is initially generated with excess energy, thereby populating higher vibrational states of low frequency modes in the complex, which by anharmonic coupling led to a red wing in the 2065 cm^−1^ CN stretching band. The observed time constant of 20 ps for its decay is typical for cooling of a vibrationally hot molecule in a solvent.^[^ ^23^ ^]^ Then, on an even slower timescale, the two marker bands of the ET_1_ intermediate at 2065 and 2172 cm^−1^ decay, and simultaneously, an absorption at 2154 cm^−1^ emerges. At the same time, the bleach of the LT species at 2080 cm^−1^ recovers by about 23 ± 5%. The final IR transient at 8.7 ns closely resembles the static difference spectrum, *A*_332K_ − *A*_292K_, suggesting that the initial photoinduced one‐electron IVCT along one Fe–Co edge of the square PBA **2** is followed by a second electron transfer step involving the other [Fe^II^_LS_Co^III^_LS_] pair. Exponential fits to time traces of the corresponding IR bands demonstrate that the [Fe^III^_LS_Co^II^_HS_]_2_ product, viz., the HT species, is formed within *τ*_1_ = 900 ± 100 ps (see Figure 5c). However, a closer comparison of the decaying 2065 and 2172 cm^−1^ bands with the static difference spectrum reveals that this transformation, even at 8.7 ns, is incomplete by leaving about 10 ± 2% of the population in ET_1_. Combined with the observed partial recovery (23%) of the bleached LT population, this suggests a relaxation kinetics involving the two equilibria LT ⇄ ET_1_ ⇄ HT. When perturbed by shuffling population from LT to ET_1_ using an 800 nm light pulse, the ET_1_ ⇄ HT equilibrium (which is preferentially on the HT side) appears to respond faster than the LT ⇄ ET_1_ equilibrium.

**Figure 6:**
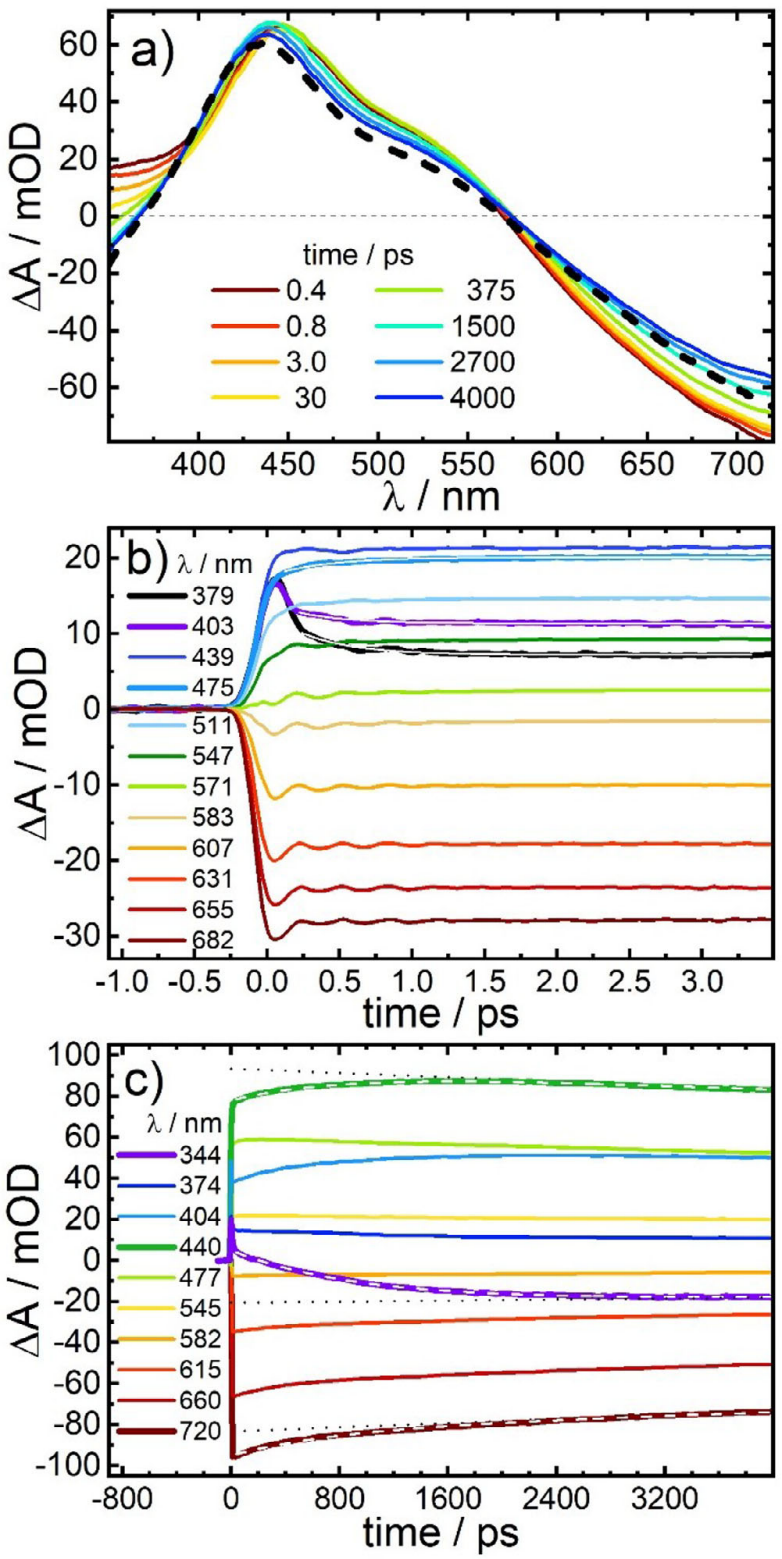
a) 800 nm pump pulse‐induced UV–vis transient difference spectra of the LT phase of PBA **2** in acetonitrile at 298 K; the black dashed line represents a scaled difference spectrum (ε_323K_ − ε_293K_) calculated from the static absorption spectra of Figure 3a. In panel b) and c) time traces are shown for selected probe wavelengths visualising the short and longtime dynamics, respectively. In (b), damped coherent oscillations with a period of 310 fs are detectable in the range 540–700 nm. For selected wavelength <500 nm, time traces are fitted by exponential decays (white lines) with time constants of 360 ± 40 fs. In c), time traces at 344, 440 and 720 nm are superimposed by double‐exponential fits (white dashed lines), yielding time constants of τ_1_ = 900 ± 60 ps and τ_2_ = 40 ± 8 ns (the τ_2_ component is represented by black dotted lines).

Our experimental findings in the UV–vis spectral range are essentially consistent with this interpretation. Figure 6a shows transients of PBA **2** after 800 nm excitation of the LT species at 298 K. Parallel to an instantaneous bleach of the IVCT transition, a positive absorption band at 450 nm with shoulders at 370 and 530 nm arises. The short time dynamics (Figures 6b, S14, and S15) are characterized by a fast blueshift of the 450 nm band and partial decay of the 370 nm shoulder with a time constant of *τ*_0_ = 360 ± 40 fs, as well as by damped coherent oscillations at probe wavelengths 540–700 nm (oscillation period *T*_osc _= 310 fs, damping constant *τ*_d_ = 350 fs). As the coherent oscillations only appear at wavelengths overlapping with the blue edge of the IVCT absorption band, we attribute these to a ground state vibrational wavepacket excited by impulsive stimulated Raman scattering (ISRS) of an intramolecular Raman active ∼108 cm^−1^ mode of the molecule.^[^ ^24^, ^25^ ^]^ Our DFT calculations show indeed a Raman active band at 106.5 cm^−1^ corresponding to the totally symmetric breathing mode of the Fe_2_Co_2_ square of the LT species including all four capping ligands. The photoinduced changes in bond lengths between metal centres and ligands effectively couple this mode to the IVCT transition (see Supporting Information for the Raman spectrum and a visualisation of the 106.5 cm^−1^ mode of LT as well as equilibrium bond lengths around the metal centres for LT and ET_1_). The spectral evolution associated with *τ*_0_ is remarkably similar to that observed for PBA **1** where a corresponding 400 fs component was assigned to SCO at the Co^II^ ion in the photoexcited Fe–Co unit, transforming Co^II^_LS_ into Co^II^_HS_.^[^ ^12^ ^]^

Up to about 60 ps, two additional time constants of 2.7 and 20 ps are required for fitting the absorption decays at wavelengths <360 nm (for details see Figure S16). Analogous to the interpretation of our time resolved IR data, we attribute these to vibrational relaxation: Figure 3a shows a steep increase of the extinction coefficient at <350 nm for both LT and HT pointing to electronic transitions with high oscillator strength at shorter wavelengths, which most likely also exist for ET_1_. As the 800 nm pump pulse and subsequent SCO produces ET_1_ with excess vibrational energy (vide infra), these transitions are initially broadened causing enhanced absorbance at their low energy side in the 350 nm region.^[^ ^23^ ^]^ During thermal equilibration, this enhanced absorbance decays. The fast component might indicate a nonstatistical energy distribution created by a preferential population of those vibrational modes that couple to the CT transition. The timescale of 2.7 ps is consistent with intramolecular vibrational redistribution (IVR) to establish a quasi‐equilibrium of the internal energy,^[^ ^26^, ^27^, ^28^ ^]^ whereas the 20 ps component is typical for thermal equilibration with the solvent.

At about 60 ps, a product spectrum emerges, which at wavelengths <400 nm differs significantly from the scaled difference (ε_323K_ − ε_293K_) of the stationary UV–vis absorption spectra at 323 and 293 K (black dashed line). In view of our IR results, we attribute this discrepancy to the formation of the ET_1_ intermediate produced by photoinduced electron transfer in only one Fe–Co unit of PBA **2**, which has a stronger absorbance at <400 nm than the HT species. In accordance with the kinetics observed for the CN stretching bands, the major part of this enhanced absorption disappears with a time constant of *τ*_1_ = 900 ± 60 ps (see, e.g., time trace at 344 nm with superimposed exponential fit of the form *b*_1_exp (− *t*/τ_1_) + *b*_2_exp (− *t*/τ_2_) (white dashed line) in Figure 6c).

The *τ*_1_ component is also responsible for 12.5% of the recovery of the bleached LT spectrum at 720 nm, consistent with the proposed LT ⇄ ET_1_ ⇄ HT equilibrium, which allows for repopulation of LT parallel to HT formation. This observation suggests that ET_1_ exhibits no absorption at 720 nm. Otherwise, the absorption signal at this wavelength is expected to decrease during the ET_1_ to HT conversion that dominates this stage of the kinetics. A similar feature was previously found for PBA **1**, indicating that for both PBAs the Fe^II^ → Co^III^ IVCT transition of the remaining Fe^II^Co^III^ pair in ET_1_ is shifted to higher energies compared to LT.^[^ ^12^ ^]^ Thus, the UV–vis spectrum of ET_1_ can be calculated from the sum of an early transient (e.g., at 60 ps, Δ*A*_60*ps*_, when vibrational relaxation is finished) and the absorption spectrum of the LT species, ε_ET1_ = *c*_1_  · Δ*A*_60ps_ + ε_LT_, where the scaling factor *c*_1_ is adjusted in such a way that the ET_1_ spectrum converges to zero for *λ* > 700 nm. The result for ε_ET1_ is presented in Figure 3a by a dashed line.

The major part of the LT recovery (87.5%) at 720 nm takes place on a significantly longer time scale (time constant *τ*_2_ = 40 ± 8 ns) after the faster ET_1_ ⇄ HT equilibrium is settled. In Figure 6c, the onset of this process is seen at pump‐probe delays >2400 ps. At this stage of the relaxation, UV–vis transients in Figure 6a assume almost the shape of the stationary difference spectrum.

**Figure 7:**
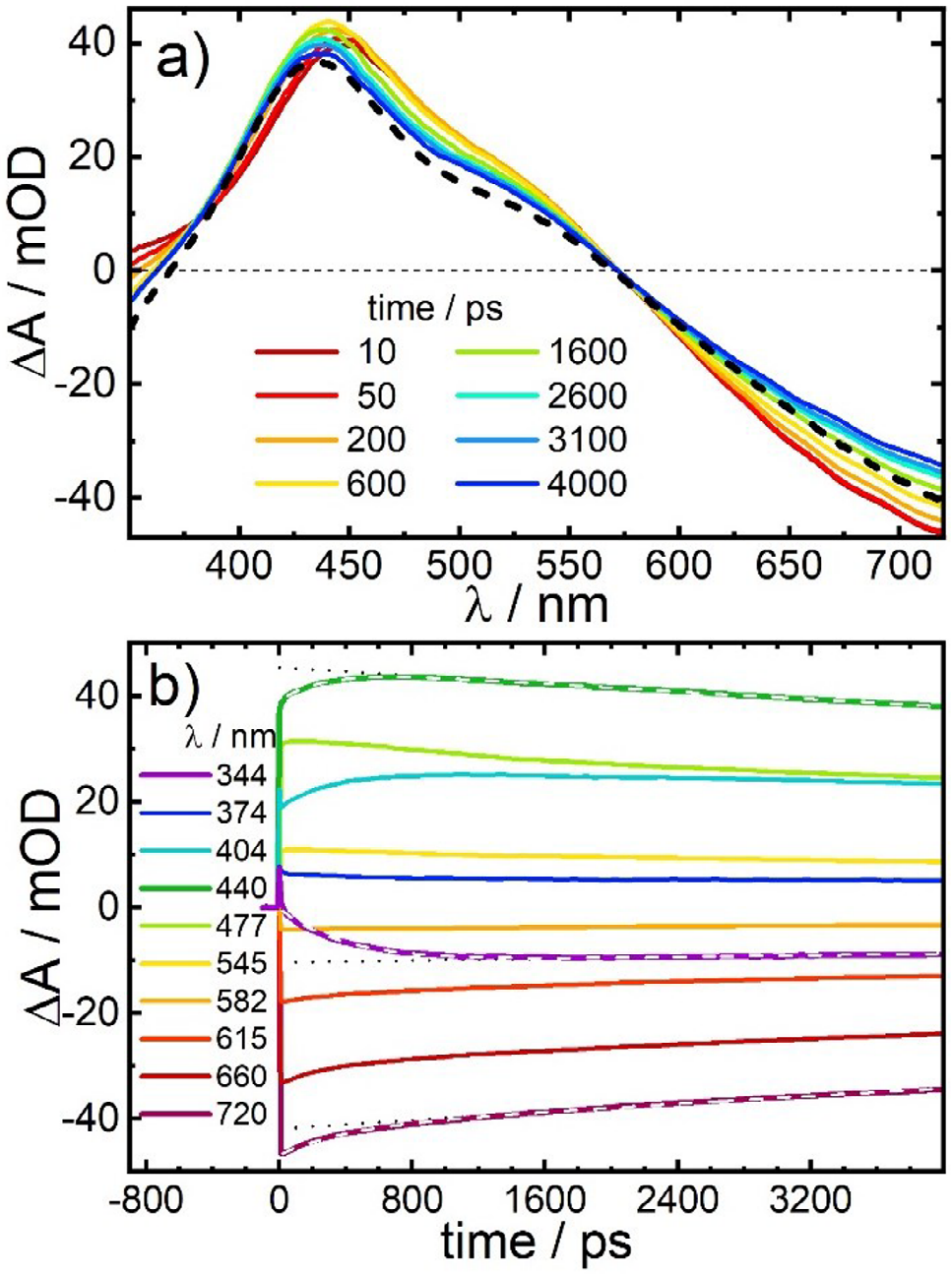
a) 800 nm pump pulse‐induced UV–vis transient difference spectra of the LT phase of PBA **2** in acetonitrile at 318 K; the black dashed line represents a scaled difference spectrum (ε_323K_ − ε_293K_) calculated from the static absorption spectra of Figure 3. b) Time traces for selected probe wavelengths (signals at 344, 440 and 720 nm are superimposed by double‐exponential fits (white dashed lines), yielding time constants of τ_1_ = 350 ± 40 ps and τ_2_ = 19 ± 3 ns (the τ_2_ component is shown by black dotted lines).

In order to better match the observed dynamics with our experimental time window of 4 ns, the UV–vis measurements were repeated at an elevated temperature of 318 K. As expected, (see Figure 7) the relaxation kinetics becomes significantly faster, yielding time constants of *τ*_1_ = 350 ± 40 ps and *τ*_2_ = 19 ± 4 ns. These time constants contribute 10.5% and 89.5%, respectively, to the recovery of the ground state at 720 nm.

### Kinetic Modelling

**Figure 8:**
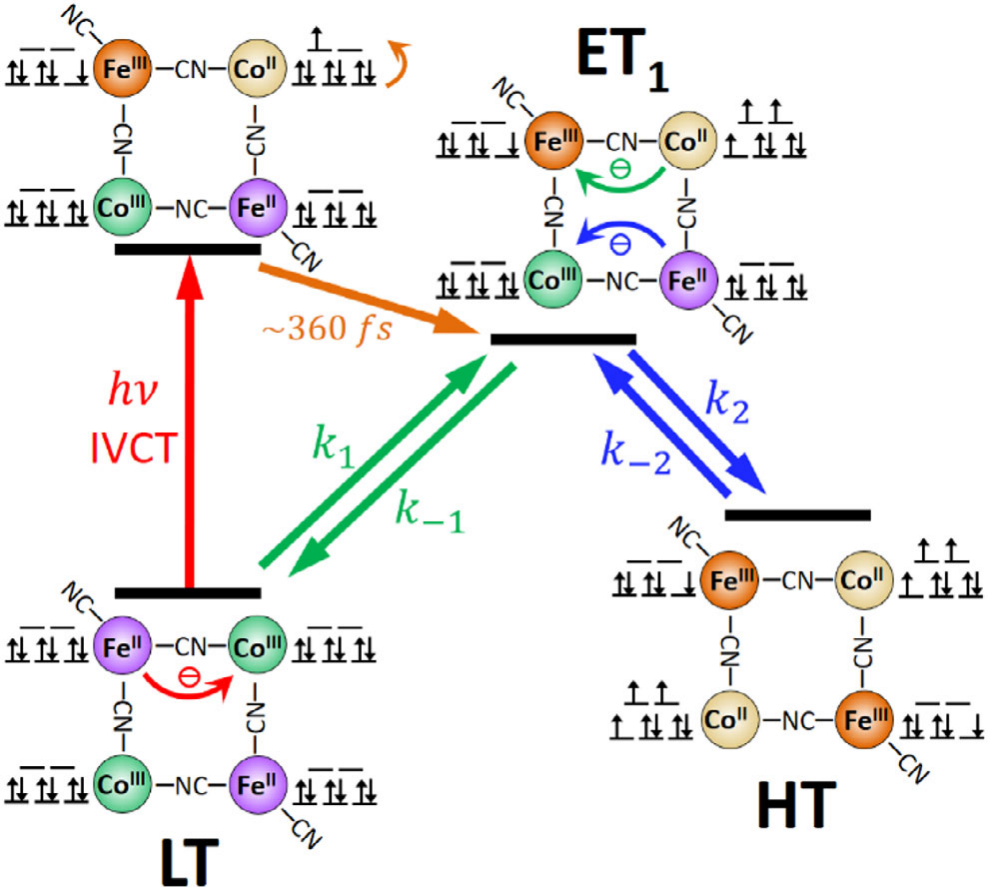
Photo‐induced cooperative electron transfer dynamics in PBA **2**. IVCT excitation of one [Fe^II^Co^III^] pair of the LT phase is followed by fast spin‐crossover, forming the [Fe^III^_LS_Co^II^_HS_Fe^II^_LS_Co^III^_LS_] intermediate (ET_1_) in about 360 fs. This disturbs the LT ⇄ ET_1_ ⇄ HT equilibrium and initiates relaxation kinetics, which transiently induce a second ETCST in the other [Fe^II^Co^III^] pair, producing a long‐lived [Fe^III^_LS_Co^II^_HS_]_2_ product (HT).

Figure 8 summarises the photoinduced electron transfer dynamics obtained so far from the analysis of our pump‐probe data. The 800 nm IVCT excitation of a [Fe^II^Co^III^] pair of the LT phase of PBA **2** is followed by spin‐crossover and produces the ET_1_ species in 360 fs, which is involved in two equilibria LT ⇄ ET_1_ and ET_1_ ⇄ HT. In this paragraph, we examine the subsequent relaxation kinetics of the disturbed LT ⇄ ET_1_ ⇄ HT equilibrium to determine the rate constants *k*_1_, *k*_−1_, *k*_2_ and *k*_−2_, as well as the associated equilibrium constants *K*_1_ = *x*_ET1_/*x*_LT_  = *k*_1_/*k*_−1_ , *K*_2_ = *x*_HT_/*x*_ET1_  = *k*_2_/*k*_−2_  and *K*  = *x*_HT_/*x*_LT_  = *K*_1_  × *K*_2_, where the *x*_i_ denote molar fractions of the involved species, LT, ET_1_ and HT. As there is no reason to believe that ET_1_ is not also present as intermediate in the thermal LT ⇄ HT transformation studied in the temperature dependent spectra of Figure 3, we will reanalyse these data to get thermodynamic constants (Δ*H* and Δ*S*) also for the individual steps of this reaction (further details of this procedure are given in Supporting Information).

Solving the coupled kinetic equations of the considered reactions results in analytical expressions for the time dependent concentration of LT, ET_1_ and HT as they approach equilibrium:

(1) $$\Delta x_{i}\left(t\right)=a_{1}\exp\left(-t/\tau_{1}\right)+a_{2}\exp\left(-t/\tau_{2}\right)$$

Here, Δ*x*_i_ is the deviation of the molar fraction of species i from its equilibrium value, and the two relaxation time constants τ_1_ and τ_2_ are functions of the four rate constants.

(2) $$\begin{array}{cc} \frac{1}{\tau_{1,2}} & =\frac{1}{2}\left(k_{1}+k_{-1}+k_{2}+k_{-2}\right)\\ & \;\pm \frac{1}{2}\sqrt{k_{1}^{2}+k_{-1}^{2}+k_{2}^{2}+k_{-2}^{2}+2k_{1}k_{-1}+2k_{2}k_{-2}+2k_{2}k_{-1}-2k_{1}k_{2}-2k_{1}k_{-2}-2k_{-1}k_{-2}} \end{array}$$

Equation (1) justifies fitting the UV–vis time traces of Figures 6c and 7b by biexponential functions. According to Equation (2), the obtained values for τ_1_ and τ_2_ represent two boundaries to determine the rate constants. Two further boundaries fixing the four unknowns, *k*_1_, *k*_−1_, *k*_2_ and *k*_−2_, are the equilibrium concentration of LT, Equation (3), taken from the UV–vis spectra of Figure 3a and the relative amplitudes of the exponential components in the recovery of the UV–vis transients at >720 nm. As only the LT species absorbs in this spectral window, the signal follows the kinetics of the low temperature species, i.e., Δ*A*_720_(*t*)∝Δ*x*_LT_(*t*), where the relative amplitudes of the exponential component *a*_1_/(*a*_1_ + *a*_2_) and *a*_2_/(*a*_1_ + *a*_2_) in Equation (1) depend on the rate constants being searched for. The input parameters used for fixing these are summarised in Table 1. The kinetic constants finally obtained are listed in Table 2.

(3) $$x_{LT}=\frac{1}{\frac{k_{1}}{k_{-1}}+\frac{k_{1}k_{2}}{k_{-1}k_{-2}}+1}\;$$

**Table 1:** Experimental parameters for determining the rate constants of the LT ⇄ ET_1_ ⇄ HT equilibrium.

$\frac{T}{K}$	τ_1_ (*ps*)	τ_2_ (*ns*)	$\frac{a_{1}}{a_{1}+a_{2}}\;\left(\%\right)$ ^a)^	*x*_LT_ (%)^b)^
298	900 ± 60	40 ± 8	12.5 ± 0.5	67.9 ± 1
318	350 ± 40	19 ± 3	10.5 ± 0.5	25.5 ± 1

^a)^ Relative amplitude of the τ_1_ component in the recovery of LT at λ>720 nm.

^b)^ Molar fraction of LT at equilibrium.

**Table 2:** Rate constants for the LT ⇄ ET_1_ ⇄ HT reaction.

	Rate constants (10^−5^ ps^−1^)			
$\frac{T}{K}$	*k*_1_	*k*_−1_	*k*_2_	*k*_−2_
298	0.91 ± 0.15	15.1 ± 1.3	86 ± 6	12.5 ± 2
318	4.4 ± 0.6	30.4 ± 1.5	244 ± 30	12.6 ± 2

**Figure 9:**
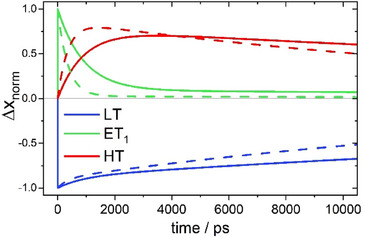
Calculated relaxation kinetics after transferring population from LT to ET_1_ at 298 K (full lines) and 318 K (dashed lines) using the rate constants of Table 2.

Based on these rate constants, the resulting relaxation kinetics after transferring population from the LT species to ET_1_ is presented in Figure 9. Of course, all the Δ*x*_i_(*t*) curves fulfill the boundaries imposed by the parameters in Table 1. In addition, the 298 K calculation for Δ*x*_ET1_(*t*) and Δ*x*_LT_(*t*) (green and blue solid lines) results in a remaining ET_1_ population of 8% and LT repopulation of 29% at *t* = 8.7 ns, which is in good agreement with the values derived from the experimental transient IR data (10 ± 2% and 23 ± 5%, respectively). Note, that in the course of the relaxation, the HT population transiently reaches 70% or 80% at 298 and 318 K, respectively, caused by the fast response of the ET_1_ ⇄ HT reaction, before the coupling to the slower LT ⇄ ET_1_ equilibrium ensures its decline.

The two data sets at 298 and 318 K allow estimating reaction and activation enthalpies and entropies for both reactions (see Tables 3 and 4). The values Δ*H* = 75 ± 7 kJ·mol^−1^ and Δ*S* = 245 ± 15 J·mol^−1^K^−1^ for the two‐electron transfer reaction are in good agreement with those obtained in our preliminary van't Hoff analysis of the temperature dependent UV–vis spectra (Δ*H* = 74 ± 7 kJ·mol^−1^, Δ*S* = 240 ± 15 J·mol^−1^K^−1^; vide supra), neglecting the ET_1_ intermediate.

**Table 3:** Thermodynamic parameters of the LT ⇄ ET_1_ ⇄ HT equilibrium.

	Δ*H* (kJ mol^−1^)	Δ*S* (J mol^−1^K^−1^)
*K*_1_	34 ± 5	92 ± 10
*K*_2_	41 ± 6	153 ± 20
*K* ^a)^	75 ± 7	245 ± 15

^a)^ *K*  = *K*_1_ *K*_2_, hence Δ*H*  = Δ*H*_1_  + Δ*H*_2_ and Δ*S*  = Δ*S*_1_  + Δ*S*_2_

**Table 4:** Activation enthalpies and entropies for the reactions LT $\overset{k_{-1}}{\leftarrow }$ ET_1_ $\overset{k_{2}}{\to }$ HT.

	Δ*H*‡ (kJ mol^−1^)	Δ*S*‡ (J mol^−1^K^−1^)
*k*_−1_	25 ± 8	−5 ± 10
*k*_2_	39 ± 8	56 ± 10

**Figure 10:**
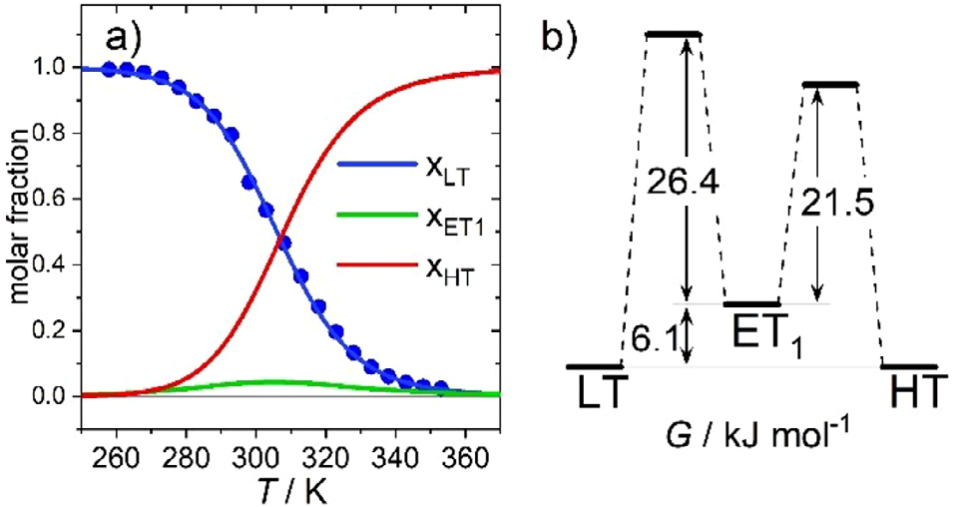
a) Molar fractions of LT, ET_1_ and HT at equilibrium. Blue points represent experimental *x*_LT_ values derived from the UV–vis spectra of Figure 3; solid lines result from calculations using the thermodynamic parameters of Table 3. Note, that the population of ET_1_ never exceeds 4.5%. b) Free energy landscape for the LT ⇄ ET_1_ ⇄ HT reaction system at *T*_½_ = 307 K (for comparison, the energy of the 800 nm photon used to excite the LT species corresponds to 150 kJ·mol^−1^).

Knowing *K*_1_(*T*) and *K*_2_(*T*) enables calculating the temperature dependent equilibrium populations of LT, ET_1_ and HT. As shown in Figure 10a, during the LT ⇄ HT transformation, the ET_1_ population never exceeds 4.5%. At the same time, the spectral signatures of ET_1_ either resemble and overlap with those of the other species or have low absorption cross sections. Taken together, this explains why ET_1_ cannot be detected in the steady‐state spectra shown in Figure 3.

Figure 10b shows the free energy landscape at the transition temperature, *T*_½_ = 307 K, determined from our data set of rate constants. At this temperature, there are equal fractions of LT and HT (i.e., Δ*G* = 0). The free energy of ET_1_ is 6.1 kJ·mol^−1^ above this level. When ET_1_ is populated, the second electron transfer occurs to give HT because the free energy barrier from ET_1_ to HT (21.5 kJ·mol^−1^) is significantly lower than for back‐electron transfer to LT (26.4 kJ·mol^−1^).

## Discussion and Conclusions

The short‐time dynamics of photoinduced ETCST for the LT species of PBA **2** is very similar to the one previously observed for PBA **1**.^[^ ^12^ ^]^ It is evident that in both cases excitation of the intense IVCT transition at 800 nm induces direct Fe→Co electron transfer in one edge of the square complex. Starting from the singlet ground state, this results in a short‐lived (360 fs) singlet [Fe^III^_LS_Co^II^_LS_Fe^II^_LS_Co^III^_LS_] species (see Figure 8), which by spin‐crossover stabilises to the [Fe^III^_LS_Co^II^_HS_Fe^II^_LS_Co^III^_LS_] product, ET_1_.^[^ ^12^ ^]^

The SCO of PBA **2** is associated with a blueshift of the 450 nm transient absorption band and simultaneous fast blueshift of the initially broad CN stretching feature at 2140 cm^−1^ towards a narrow band at 2172 cm^−1^. Quite similar spectral evolutions were observed also during SCO in PBA **1** on an almost identical timescale (400 fs).^[^ ^12^ ^]^

Although in PBA **1** the photoinduced electron transfer was restricted to only one Fe–Co unit of the square complex (at least in the time window up to 1.5 ns), in PBA **2** a second electron transfer step is triggered at the opposite edge of the [Fe_2_Co_2_] square, as shown by the agreement between the 8.7 ns transient IR spectrum and the scaled difference spectrum (*A*_332K_ − *A*_292K_) in Figure 5b. The disappearance of the 2065 and 2172 cm^−1^ marker bands of ET_1_ indicates that a single photon eventually induces two ETCST events. Cooperative photoswitching was previously observed for crystalline PBA samples inside the LT ⇄ HT hysteresis loop when irradiated with high intensity laser pulses.^[^ ^14^, ^15^ ^]^ Above an absorbed photon density corresponding to >50% of the excited [Fe^II^Co^III^] units, complete LT→HT conversion was achieved by a single laser pulse.^[^ ^15^ ^]^ From the mechanistic point of view, it is not clear, however, whether the observed cooperativity was due to elastic coupling induced by lattice strain of the primary absorbing and transformed [Fe^II^Co^III^] units or due to thermal heating of the crystal above the threshold temperature of the LT→HT transition.

This issue was elaborated in greater detail for [Fe(phen)_2_(NCS)_2_] spin‐crossover crystals. Bertoni et al.^[^ ^17^ ^]^ demonstrated that a local phototriggered LS‐to‐HS transition causes lattice distortions which induce additional LS‐to‐HS switching in a ns time window. Subsequently, a further increase of the HS fraction is observed within several µs due to thermal heating of the crystal.

In our experiments, the molecular PBA **2** is diluted in MeCN solution and was excited with pulse energies of 1 µJ absorbed in a volume of 0.02 mm^3^. If transformed into heat, this energy leads to a maximum temperature rise of 0.04 K, which is too small to induce a thermal ETCST. Also, a scenario where part of the absorbed photon generates transiently a vibrationally hot complex which undergoes a thermal ETCST can be ruled out because cooling of such a species in solution is significantly faster (∼20 ps) than the observed time constant of *τ*_1_ = 900 ± 100 ps for the second ETCST step. Instead, our kinetic analysis clearly shows that the observed cooperative switching in PBA **2** is caused by two coupled equilibria favouring the second electron transfer when the first is induced either thermally or by light absorption. Additional studies for PBA 1 covering timescales up to microseconds would be useful to clarify whether the two PBAs really behave qualitatively different.

The question arises as to the mechanism triggering the second ETCST step in the ET_1_ state. Although this may tentatively be attributed to structural strain in the less symmetrical [Fe^II^_LS_Co^III^_LS_Fe^III^_LS_Co^II^_HS_] square and a more favourable charge distribution in the symmetrical LT and HT species (where the 3+ metal ions are at opposite corners of the square), the experimental data and electronic structure calculations support that the process is driven by entropy. Both conversions, LT→ET_1_ and ET_1_→HT, are enthalpically disfavoured, with roughly the same enthalpic penalty (Table 3). However, they are driven entropically by 92 and 153 J·mol^−1^K^−1^), respectively; based on the rigid‐rotor harmonic approximation, our calculations give values of 71 and 104 J·mol^−1^K^−1^ (note that the underestimation is expectable as we are unable to calculate the entropy change in the solvent). This entropy change is predominantly rooted in the vibrational term, and the entropic driving force is significantly larger for the second step, ET_1_→HT. As the number of unpaired electrons is increased, the metal–ligand bonds become less constrained. Particularly, in the HT case, the complex exhibits lower energy (delocalised) vibrational modes, whereas there is still some constraint in the ET_1_ state, given that only one side has been excited. It will be interesting to investigate whether this entropic differentiation between the LT→ET_1_ and ET_1_→HT steps holds for other square PBAs as well, and how it is possibly correlated with their structures.

Although cooperative effects in stimuli–responsive molecule‐based spin transition materials have so far mostly been considered as short‐ and long‐range elastic intermolecular interactions in the solid state,^[^ ^29^ ^]^ in this work we report unprecedented intramolecular cooperativity between the two Fe–Co constituent subunits of a cyanide‐bridged square Fe_2_Co_2_ Prussian blue analogue in solution. Inducing two sequential ETCST events by a single photon to eventually give a doubly switched long‐lived metastable entity demonstrates how cooperativity on a molecular level can lead to extended lifetimes of optically excited charge transfer and spin states in coupled oligometallic systems, which we believe holds great potential for the design of molecule‐based photoresponsive switching devices.

## Supporting Information

The Supporting Information contains details of the synthesis and characterization; additional spectroscopic and magnetic data; information on the solution properties and thermal decomposition of **2**; details of the pump‐probe experiments and the kinetic analysis; and computational details with Cartesian coordinates. The authors have cited additional references within the Supporting Information.^[^ ^30^, ^31^, ^32^, ^33^, ^34^, ^35^, ^36^, ^37^, ^38^, ^39^, ^40^, ^41^ ^]^

## Conflict of Interests

The authors declare no conflict of interest.

## Supporting information



Supporting Information

Supporting Information

## Data Availability

The data that support the findings of this study are available from the corresponding author upon reasonable request.

## References

[1] J. Yadav , R. Kharel , S. Konar , Coord. Chem. Rev. 2025, 523, 216283.

[2] D. Aguilà , Y. Prado , E. S. Koumousi , C. Mathonière , R. Clérac , Chem. Soc. Rev. 2016, 45, 203–224. 26553752 10.1039/c5cs00321k

[3] M.‐G. Alexandru , D. Visinescu , J. Cano , F. Lloret , M. Julve , Cryst. Growth Des. 2023, 23, 1288–1308.

[4] T. J. Penfold , J. O. Johansson , J. Eng , Coord. Chem. Rev. 2023, 494, 215346.

[5] L. Jean‐François , G. Philippe , G.‐C. Laurence , Top. Curr. Chem. 2004, 235, 221–249.

[6] P. Gamez , J. Sánchez Costa , M. Quesada , G. Aromí , Dalton Trans. 2009, 7845–7853. 19771343 10.1039/b908208e

[7] M. Nihei , Y. Sekine , N. Suganami , K. Nakazawa , A. Nakao , H. Nakao , Y. Murakami , H. Oshio , J. Am. Chem. Soc. 2011, 133, 3592–3600. 21341801 10.1021/ja109721w

[8] S. Kamilya , S. Ghosh , Y. Li , P. Dechambenoit , M. Rouzières , R. Lescouëzec , S. Mehta , A. Mondal , Inorg. Chem. 2020, 59, 11879–11888. 32803968 10.1021/acs.inorgchem.0c02053

[9] T. Ikeda , Y.‐B. Huang , S.‐Q. Wu , W. Zheng , W.‐H. Xu , X. Zhang , T. Ji , M. Uematsu , S. Kanegawa , S.‐Q. Su , O. Sato , Dalton Trans. 2024, 53, 15465–15470. 39239808 10.1039/d4dt01581a

[10] K. Barlow , J. O. Johansson , Phys. Chem. Chem. Phys. 2021, 23, 8118–8131. 33875986 10.1039/d1cp00535a

[11] M. Cammarata , S. Zerdane , L. Balducci , G. Azzolina , S. Mazerat , C. Exertier , M. Trabuco , M. Levantino , R. Alonso‐Mori , J. M. Glownia , S. Song , L. Catala , T. Mallah , S. F. Matar , E. Collet , Nat. Chem. 2021, 13, 10–14. 33288895 10.1038/s41557-020-00597-8

[12] J. Zimara , H. Stevens , R. Oswald , S. Demeshko , S. Dechert , R. A. Mata , F. Meyer , D. Schwarzer , Inorg. Chem. 2021, 60, 449–459. 33332100 10.1021/acs.inorgchem.0c03249

[13] J. Yadav , D. J. Mondal , S. Konar , Chem. Commun. 2021, 57, 5925–5928. 10.1039/d1cc01160j 34013926

[14] H. W. Liu , K. Matsuda , Z. Z. Gu , K. Takahashi , A. L. Cui , R. Nakajima , A. Fujishima , O. Sato , Phys. Rev. Lett. 2003, 90, 167403. 12732007 10.1103/PhysRevLett.90.167403

[15] N. Shimamoto , S. Ohkoshi , O. Sato , K. Hashimoto , Inorg. Chem. 2002, 41, 678–684. 11849066 10.1021/ic010915u

[16] R. Bertoni , M. Cammarata , M. Lorenc , S. F. Matar , J.‐F. Létard , H. T. Lemke , E. Collet , Acc. Chem. Res. 2015, 48, 774–781. 25705921 10.1021/ar500444d

[17] R. Bertoni , M. Lorenc , T. Graber , R. Henning , K. Moffat , J.‐F. Létard , E. Collet , CrystEngComm 2016, 18, 7269–7275. 28127256 10.1039/C6CE00659K PMC5256688

[18] J. de Jesus Velazquez‐Garcia , K. Basuroy , D. Storozhuk , J. Wong , S. Demeshko , F. Meyer , R. Henning , S. Techert , Dalton Trans. 2022, 51, 17558–17566. 36315244 10.1039/d2dt02638d PMC9749069

[19] E. Ramos‐Cordoba , V. Postils , P. Salvador , J. Chem. Theory Comput. 2015, 11, 1501–1508. 26574361 10.1021/ct501088v

[20] M. Gimferrer , J. Van der Mynsbrugge , A. T. Bell , P. Salvador , M. Head‐Gordon , Inorg. Chem. 2020, 59, 15410–15420. 33030893 10.1021/acs.inorgchem.0c02405

[21] M. Gimferrer , S. Danés , D. M. Andrada , P. Salvador , Inorg. Chem. 2021, 60, 17657–17668. 34766771 10.1021/acs.inorgchem.1c02252 PMC8653152

[22] P. Salvador , E. Ramos‐Cordoba , M. Montilla , L. Pujal , M. Gimferrer , J. Chem. Phys. 2024, 160, 172502. 38748009 10.1063/5.0206187

[23] D. Schwarzer , J. Troe , M. Votsmeier , M. Zerezke , J. Chem. Phys. 1996, 105, 3121–3131.

[24] H. Kuramochi , T. Tahara , J. Am. Chem. Soc. 2021, 143, 9699–9717. 34096295 10.1021/jacs.1c02545 PMC9344463

[25] A. B. Myers , Chem. Rev. 1996, 96, 911–926. 11848775 10.1021/cr950249c

[26] D. Schwarzer , C. Hanisch , P. Kutne , J. Troe , J. Phys. Chem. A 2002, 106, 8019–8028.

[27] D. Schwarzer , P. Kutne , C. Schröder , J. Troe , J. Chem. Phys. 2004, 121, 1754–1764. 15260725 10.1063/1.1765092

[28] B. Schluschaß , J.‐H. Borter , S. Rupp , S. Demeshko , C. Herwig , C. Limberg , N. A. Maciulis , J. Schneider , C. Würtele , V. Krewald , D. Schwarzer , S. Schneider , JACS Au 2021, 1, 879–894. 34240082 10.1021/jacsau.1c00117 PMC8243327

[29] Y. Fang , Y.‐S. Meng , H. Oshio , T. Liu , Coord. Chem. Rev. 2024, 500, 215483.

[30] J. Kim , S. Han , I.‐K. Cho , K. Y. Choi , M. Heu , S. Yoon , B. J. Suh , Polyhedron 2004, 23, 1333–1339.

[31] S. Lorenz , B. Plietker , ChemCatChem 2016, 8, 3203–3206.

[32] O. Kahn , Molecular Magnetism. VCH‐Verlag, USA 1993.

[33] M. Nihei , M. Ui , N. Hoshino , H. Oshio , Inorg. Chem. 2008, 47, 6106–6108. 18563896 10.1021/ic7024582

[34] J. I. Steinfeld , J. S. Francisco , W. L. Hase , Chemical Kinetics and Dynamics, Prentice Hall, New Jersey 1999.

[35] J. Franz , M. Oelschlegel , J. P. Zobel , S.‐A. Hua , J.‐H. Borter , L. Schmid , G. Morselli , O. S. Wenger , D. Schwarzer , F. Meyer , L. González , J. Am. Chem. Soc. 2024, 146, 11272–11288. 38598687 10.1021/jacs.4c00548 PMC11046484

[36] Gaussian 16, Revision C.01, M. J. Frisch , G. W. Trucks , H. B. Schlegel , G. E. Scuseria , M. A. Robb , J. R. Cheeseman , G. Scalmani , V. Barone , G. A. Petersson , H. Nakatsuji , X. Li , M. Caricato , A. V. Marenich , J. Bloino , B. G. Janesko , R. Gomperts , B. Mennucci , H. P. Hratchian , J. V. Ortiz , A. F. Izmaylov , J. L. Sonnenberg , D. Williams‐Young , F. Ding , F. Lipparini , F. Egidi , J. Goings , B. Peng , A. Petrone , T. Henderson , D. Ranasinghe , et al., Gaussian, Inc., Wallingford CT 2016.

[37] R. Li , J. Zheng , D. G. Truhlar , Phys. Chem. Chem. Phys. 2010, 12, 12697. 20733991 10.1039/c0cp00549e

[38] F. Weigend , R. Ahlrichs , Phys. Chem. Chem. Phys. 2005, 7, 3297. 16240044 10.1039/b508541a

[39] P. Salvador , E. Ramos‐Cordoba , J. Chem. Phys. 2013, 139, 071103. 23968064 10.1063/1.4818751

[40] N. S. Hush , Prog. Inorg. Chem. 1967, 8, 391–444.

[41] B. S. Brunschwig , N. Sutin , Coord. Chem. Rev. 1999, 187, 233–254.

