# Supplementary material for: Cooperativity of Electron Transfer Coupled Spin Transitions in a Tetranuclear Fe/Co Prussian Blue Analogue Revealed by Ultrafast Spectroscopy
Source: Angew Chem Int Ed Engl. 2025 May 9;64(27):e202505813. doi: 10.1002/anie.202505813 (PMC12207354; doi:10.1002/anie.202505813)
Supplement: Supplementary file 1 — Supporting Information [file ANIE-64-e202505813-s001.pdf]

Supporting Information

Cooperativity of Electron Transfer Coupled Spin Transitions in a Tetranuclear Fe/Co Prussian Blue Analogue Revealed by Ultrafast Spectroscopy

Jan-Hendrik Borter,#[a] Simindokht Gol Kar,#[b] Sayan Kangsa Banik,#[a] Serhiy Demeshko,[b] Sebastian Dechert,[b] Rainer Oswald,[c] Martí Gimferrer,[c] Ricardo A. Mata,*[c] Dirk Schwarzer,*[a] and Franc Meyer*[b]

# These authors contributed equally to this work

* Corresponding authors

[a] J.H. Borter, S. Kangsa Banik, D. Schwarzer
Max-Planck-Institute for Multidisciplinary Sciences, Department of Dynamics at Surface
Am Fassberg 11, 37077 Göttingen, Germany
Email: dschwar@mpinat.mpg.de

[b] S. Gol Kar, S. Demeshko, S. Dechert, F. Meyer
Institute for Inorganic Chemistry, Georg-August-Universität Göttingen
Tammannstrasse 4, 37077 Göttingen, Germany
E-mail: franc.meyer@chemie.uni-goettingen.de

[c] R. A. Mata, M. Gimferrer, R. Oswald
Institute for Physical Chemistry, Georg-August-Universität Göttingen
Tammannstrasse 6, 37077 Göttingen, Germany
E-mail: ricardo.mata@chemie.uni-goettingen.de

1. Contents

1. Contents 2
2. Materials and Syntheses 3
3. Magnetic Measurements of PBA **2** 3
4. ^{57}Fe Mössbauer Spectroscopy of PBA **2** 4
5. ESI Mass Spectrometry of PBA **2** 5
6. NMR Spectroscopy of PBA **2** 6
7. Thermal Decomposition of PBA **2** 7
8. Van’t Hoff Analysis of the LT ⇄ HT Equilibrium of **2** 13
9. IR Spectrum of ET_1 14
10. UV/Vis Transients and Time Traces with Exponential Fits 15
11. Determining the Rate Constants of the LT ⇄ ET_1 ⇄ HT Equilibrium 17
12. Fs-UV/Vis (TR-UV/vis) and -IR (TR-IR) Pump Probe Spectroscopy 19
13. Computational Details 20
14. Raman Active Mode Causing the Wavepacket in the UV/Vis Transients 21
15. Mulliken-Hush Analysis of the IVCT Absorption Band 23
16. Cartesian Coordinates (XYZ) of the Studied Systems 24
17. References 50

2. Materials and Syntheses

All chemicals were purchased from Sigma-Aldrich and used as received. All solvents used were at least reagent grade. Reactions were performed under ambient conditions. The building block K[Fe(Tp)$(CN)_3$] and the tetradentate N-donor ligand {en(Bn)py} were synthesized following the reported method.[1,2] The square PBA **2**, $[Fe(Tp)(CN)_3]_2[Co\{N_2Py_2\}]_2(ClO_4)_2$, was prepared as described by Yadav et al.[3]

IR spectra were recorded on an Agilent Technologies Cary 630 FTIR spectrometer with Dial Path Technology and analyzed with FTIR MicroLab software.

NMR spectra were recorded on a Bruker Avance III HD 500 spectrometer in acetonitrile-d3 (δ = 1.94 ppm), with residual solvent signals as internal references. All signals were numbered for convenient assignments and the multiplicity was abbreviated as (s) for singlet, (d) duplet, (t) triplet, (q) quartet, and (m) as multiplet. The chemical shifts (δ) are given in ppm and coupling constants in Hz. Analysis of the spectra was carried out with MestReNova version 14.2.0-26256.

A Bruker HCT Ultra Instrument was used to conduct the ESI-MS experiments. Bruker Daltonics software was used for all data collecting and processing. The ESI-MS spectra were plotted using OriginPro 2021.

3. Magnetic Measurements of PBA 2

Temperature-dependent magnetic susceptibility measurements were carried out with a Quantum-Design MPMS3 SQUID magnetometer equipped with a 7 Tesla magnet in the ranges from 300 to 400 and from 400 to 2 K at a magnetic field of 0.5 T. The powdered sample was contained in a polycarbonate capsule and fixed in a non-magnetic sample holder. Each raw data file for the measured magnetic moment was corrected for the diamagnetic contribution of the polycarbonate capsule according to $M^{dia}(\text{capsule}) = \chi_g \cdot m \cdot H$, with an experimentally obtained gram susceptibility of the polycarbonate capsule.

The molar susceptibility data were corrected for the diamagnetic contribution according to χ_M^{dia}(sample) = $-0.5 \cdot M \cdot 10^{-6}$ $cm^3 \cdot mol^{-1}$.[4]

Figure S1 shows the SQUID data for **2**·4H_2O·4MeOH with a LS→HS transition at $T_{½}$ = 374 K upon heating and a HS→LS transition at $T_{½}$ = 323 K upon cooling (where LS is $[Fe^{II}_{LS}Co^{III}_{LS}]_2$ and HS is $[Fe^{III}_{LS}Co^{II}_{HS}]_2$). The transition temperatures are strongly dependent on the solvent molecules in the crystal lattice.

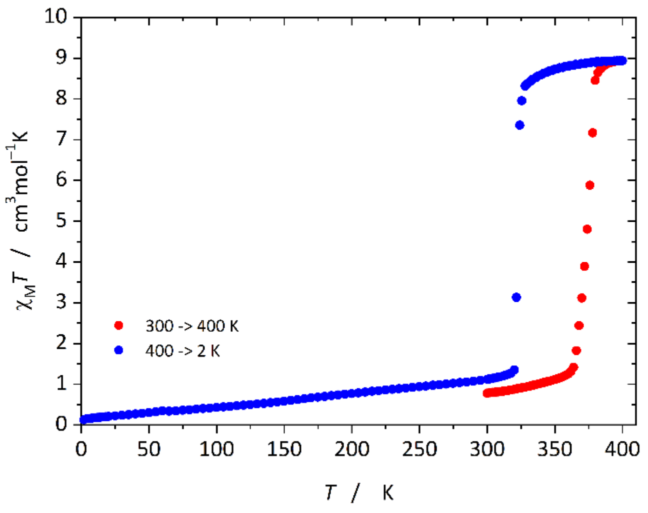

Figure S1: $\chi_M T$ vs. T plot for solid **2**·4H_2O·4MeOH in the range from 2 to 400 K

4. ^{57}Fe Mössbauer Spectroscopy of PBA 2

The Mössbauer spectrum of PBA **2** was recorded with a ^{57}Co source in a Rh matrix using an alternating constant acceleration Wissel Mössbauer spectrometer operated in the transmission mode and equipped with a Janis closed-cycle helium cryostat. Isomer shifts are given relative to iron metal at ambient temperature. Simulation of the experimental data was performed with the Mfit program using Lorentzian line doublets (E. Bill, Max-Planck Institute for Chemical Energy Conversion, Mülheim/Ruhr, Germany).

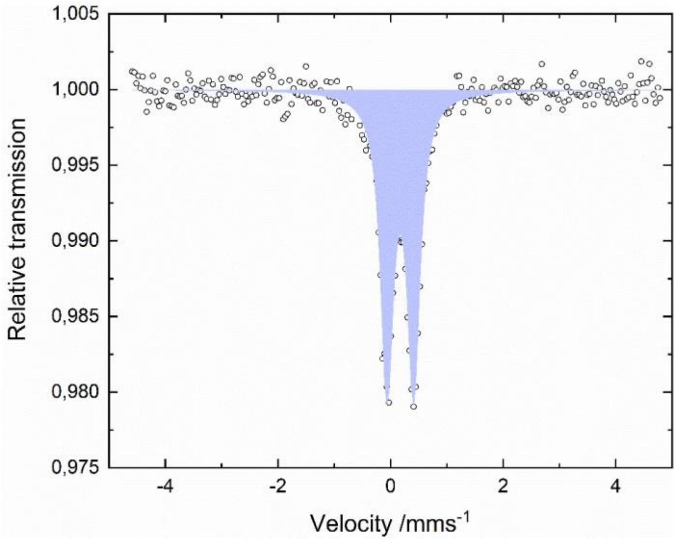

Figure S2. Zero-field ^{57}Fe Mössbauer spectrum of solid **2** at 80 K.

5. ESI Mass Spectrometry of PBA 2

The electrospray ionization (ESI) mass spectrum of PBA **2** in MeCN solution was collected on a Bruker HCT ultra instrument. The spectrum shown in Figure S3 shows one dominant peak at *m*/*z* = 828.4, corresponding to the ion $[\{Fe(Tp)(CN)_3\}_2\{Co(N_2Py_2)\}_2]^{2+}$.

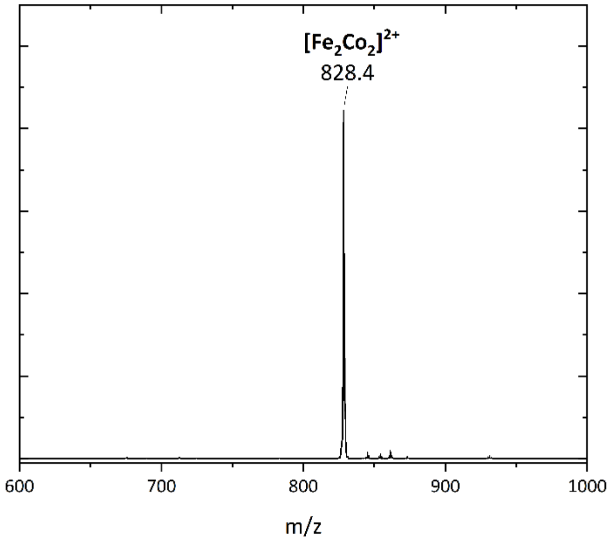

Figure S3. ESI(+) mass spectrum of **2** in MeCN solution.

6. NMR Spectroscopy of PBA 2

An ^{1}H NMR spectrum of PBA **2** in MeCN-d3 solution recorded at 253 K (Figure S11) confirms that the compound is diamagnetic at that temperature.

^{1}H NMR (600 MHz, CD_3CN): δ [ppm] = 9.39 (d, J = 5.9 Hz, 2H), 8.06 – 8.02 (m, 2H), 7.81 (td, J = 7.7, 1.3 Hz, 2H), 7.68 (d, J = 6.9 Hz, 2H), 7.61 (dd, J = 2.3, 0.7 Hz, 2H), 7.40 – 7.38 (m, 1H), 7.28 – 7.20 (m, 2H), 7.16 (t, J = 7.5 Hz, 4H), 6.91 (d, J = 7.8 Hz, 2H), 6.81 – 6.77 (m, 4H), 6.21 (d, J = 1.9 Hz, 1H), 6.04 (t, J = 2.0 Hz, 2H), 5.78 (t, J = 2.0 Hz, 1H), 3.95 (dd, J = 14.5, 8.1 Hz, 4H), 3.14 (d, J = 15.6 Hz, 2H), 3.09 (d, J = 13.2 Hz, 2H), 2.53 (t, J = 11.9 Hz, 2H), 2.40 (d, J = 10.0 Hz, 2H).

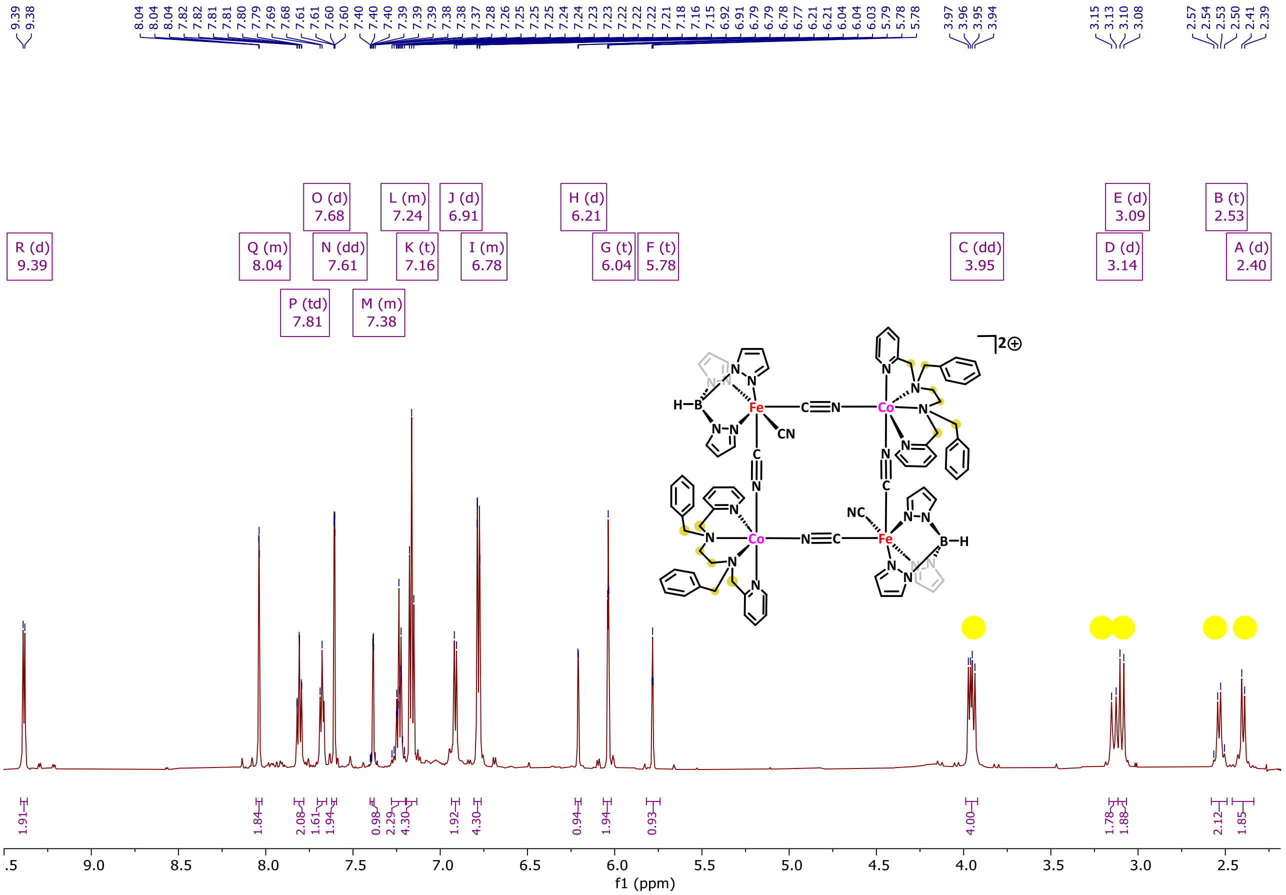

Figure S4: ^{1}H NMR spectrum of **2** in MeCN-d_3 at 253 K.

7. Thermal Decomposition of PBA 2

The thermal stability of **2** in MeCN solution has been investigated to determine the temperature range suitable for the ultrafast pump probe experiments. While temperature dependent UV/vis spectra up to 328 K show full reversibility (Figure S5, Bottom-left), reversibility is partially lost upon heating the sample of **2** in MeCN up to 353 K (Figure S5, top-left); the right side of Figure S5 shows the absorption changes at 800 nm as a function of temperature for heating and subsequent cooling cycles. Hence, all pump probe experiments were conducted at temperatures below 328 K.

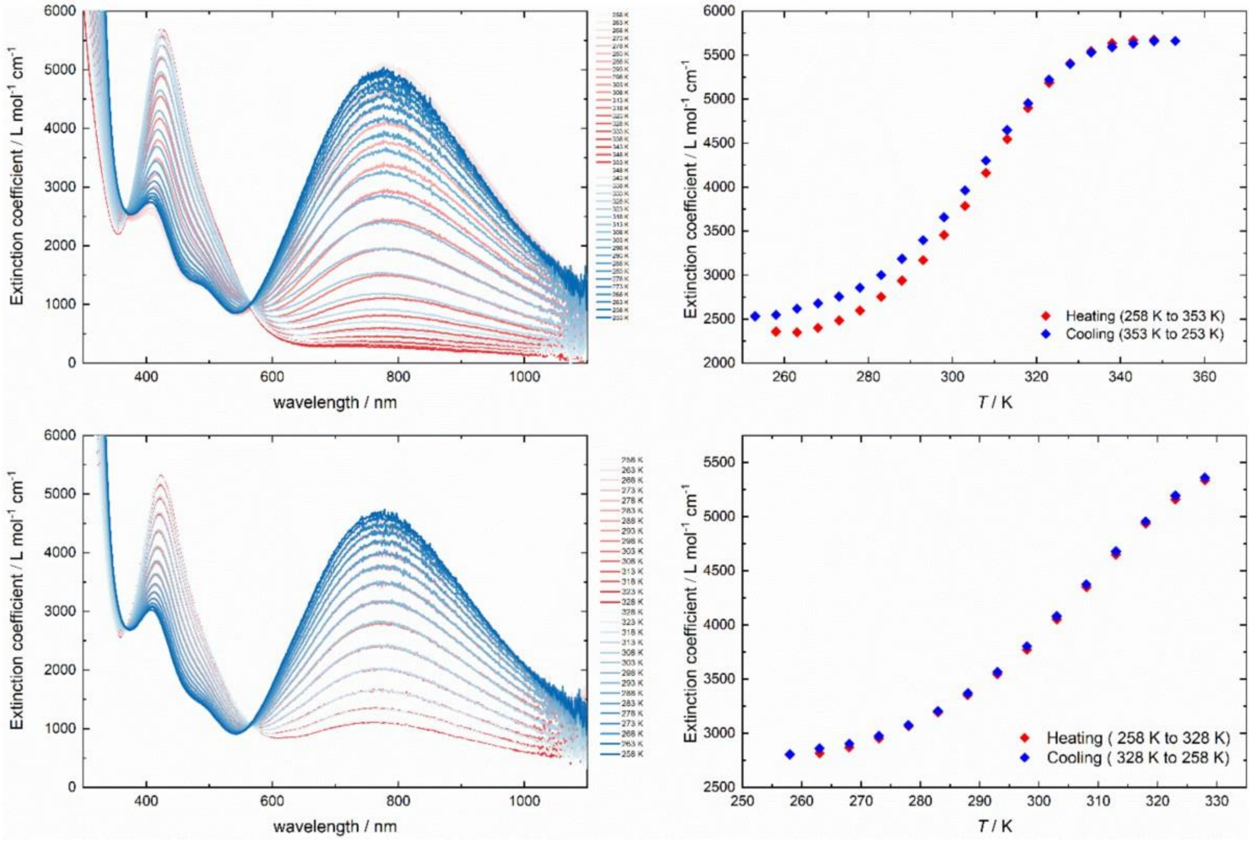

Figure S5: VT-UV/Vis spectra of 2 in MeCN solution for temperature ranges 258 K → 353 K → 253 K (top) and 258 K → 328 K → 253 K (bottom); the left side shows an overlay of all UV/Vis spectra during heating (red) and cooling cycles, while the right side shows the corresponding changes of absorption at 800 nm (top-right). VT-UV/Vis heating from 258 k to 328 K and cooling again to 253 K (bottom-left) overlaying of the absorption of both heating and cooling cycles based on the temperature-dependent UV/vis spectra at 800 nm (bottom-right).

To gain a comprehensive understanding of the behavior at higher temperatures, a more detailed investigation was undertaken. A solution of **2** in MeCN was heated to 353 K for 48 h and the conversion of **2** monitored by UV/Vis spectroscopy recorded at 2 h intervals (Figure S6). The absorption around 800 nm gradually increases, while the band at 420 nm decreases. It should be noted that crystalline **2** showed no changes when heated to 353 K for two days, indicating that solid **2** is stable at that temperature (Figure S7).

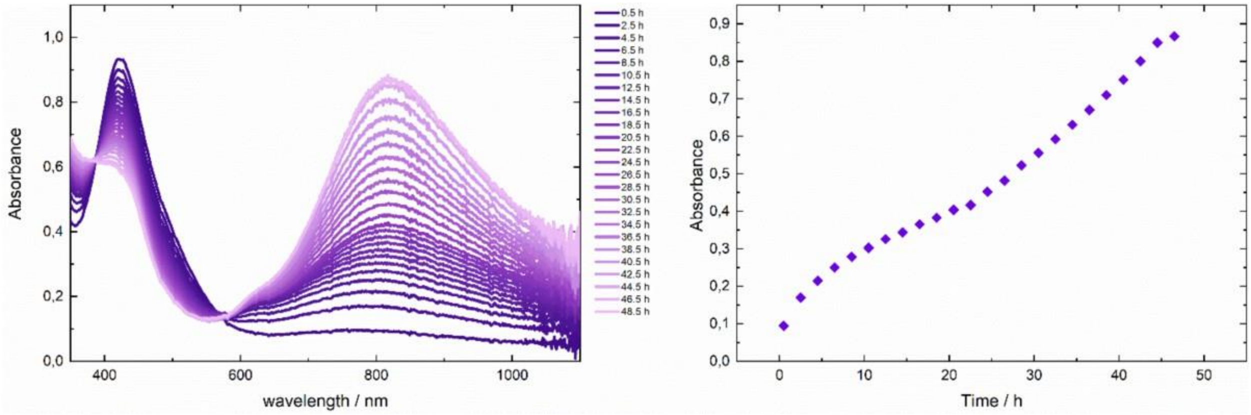

Figure S6: UV/Vis spectra of a solution of **2** in MeCN at 353 K recorded every two hours over a period of two days (left) and absorption changes at 800 nm over time (right)

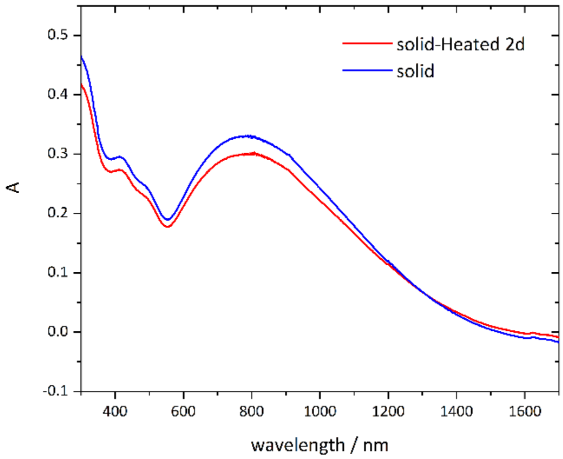

Figure S7: Diffuse reflectance UV/Vis spectra of solid **2** before and after heating the sample to 353 K for two days.

IR spectra were recorded for a fresh crystalline sample of PBA **2** and for the powder obtained after heating a sample of **2** in MeCN to 348 K for 48 h and evaporation of the solvent (Figure S8), clearly evidencing degradation of **2** (only a single broad CN stretching band at 2077 cm^{-1} is observed for the heated sample).

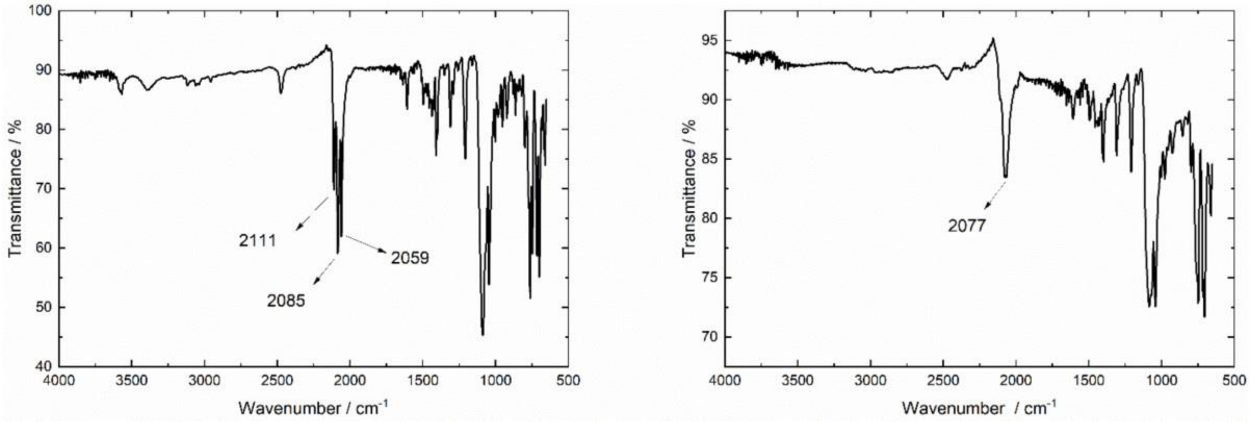

Figure S8: IR spectra of a crystalline sample of PBA **2** (left) and of the powder obtained after heating a sample of **2** in MeCN to 348 K for 48 h and evaporation of the solvent.

To identify the products of the thermal degradation of **2** in MeCN solution, crystalline material of **2** was dissolved in MeCN and then heated to 348 K for 48 h. An ESI(+) mass spectrum recorded after 48 h shows the ions $[Co(N_2Py_2)(CN)_2]^+$ and $[Fe(TP)_2]^+$ (Figure S9), suggesting that the compound has disintegrated into separate Co- and Fe-containing fragments.

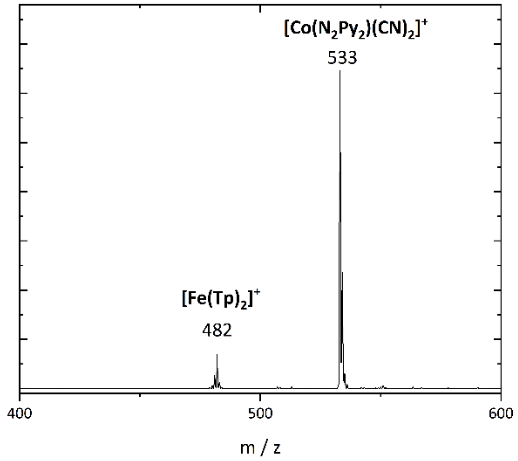

Figure S9: ESI(+) mass spectrum of a solution of **2** in MeCN after the solution of **2** was heated to 348 for 48 h.

The ^{1}H NMR spectrum of a solution of **2** in MeCN-d_3 that was heated to 348 K for 48 h (Figure S10) clearly differs from the spectrum of genuine **2** (cf. Figure S4). While Figure S10 shows signals that can be assigned to the ligand N_2Py_2, signals for the Tp ligand are missing, which in combination with the ESI-MS results suggests that **2** has disintegrated into a diamagnetic Co^{III} complex containing the ligand N_2Py_2 and a paramagnetic complex containing the {(Tp)Fe} fragment (see below). DOSY experiments of a sample of **2** in MeCN-d_3 recorded before and after heating (Figure S11) provide diffusion coefficients D = 2.6×10^{-10} m^2/s for genuine **2** and D = 4×10^{-10} m^2/s for the diamagnetic component of the heated sample, indicating that the volume V_{heated} of the diamagnetic component is roughly ¼ of the volume V_{PBA} of **2**, hence that the diamagnetic component in the heated sample is a mononuclear complex.

$$D = \frac{KT}{6\pi\eta r} \qquad D \sim \frac{1}{r} \sim \frac{1}{\sqrt[3]{V}} \qquad \frac{V_{PBA}}{V_{heated}} \sim 3.7$$

where r is the radius of the respective fragment.

The combined ESI-MS, ^{1}H NMR and DOSY experiments suggest that the diamagnetic decomposition product that results after heating **2** to 348 K for 48 h is the mononuclear complex cation $[Co^{III}(N_2Py_2)(CN)_2]^+$. We propose that its formation is caused by rotation of the cyanido ligands, which are C-bound to Fe in **2** but C-bound to Co after heating.

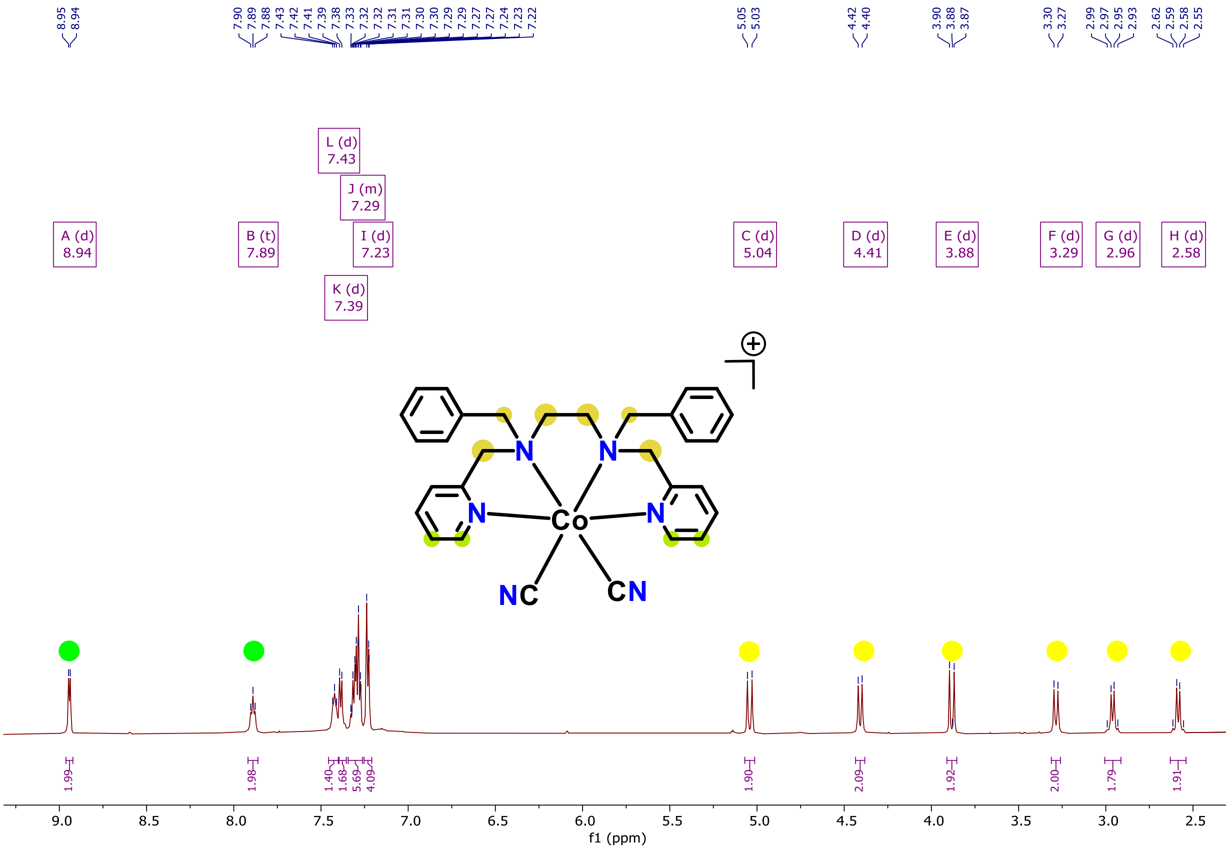

Figure S10: ^{1}H NMR spectrum of a solution of **2** in MeCN-d_3 after the solid sample of **2** was heated to 348 K for 48 h.

^{1}H NMR (600 MHz, CD_3CN) δ [ppm] = 8.94 (d, *J* = 5.7 Hz, 2H), 7.89 (t, *J* = 7.8 Hz, 2H), 7.43 (d, *J* = 6.5 Hz, 2H), 7.39 (d, *J* = 7.7 Hz, 2H), 7.34 – 7.26 (m, 6H), 7.23 (d, *J* = 6.8 Hz, 4H), 5.04 (d, *J* = 16.0 Hz, 2H), 4.41 (d, *J* = 13.4 Hz, 2H), 3.88 (d, *J* = 16.0 Hz, 2H), 3.29 (d, *J* = 13.4 Hz, 2H), 2.96 (d, *J* = 10.3 Hz, 2H), 2.58 (d, *J* = 10.2 Hz, 2H).

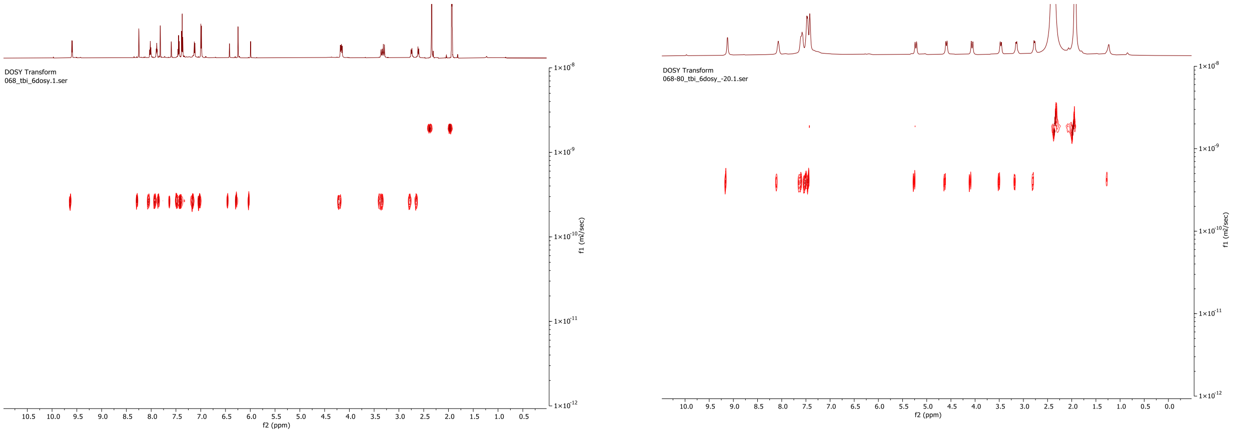

Figure S11. DOSY experiments at 253 K in MeCN-d_3 of the genuine complex **2** (left) and after heating 2 to heated sample (right)

After several weeks, the formation of few crystals was observed in the NMR tube containing the sample of **2** that was heated to 348 K for 48 h in MeCN-d_3. X-ray crystallographic analysis revealed that the known[5] mixed-valent cubic complex $[(Tp)_8Fe^{III}{}_4Fe^{II}{}_4(CN)_{12}]$ was formed (a = b = c = 33.722(4) Å, $\alpha = \beta = \gamma = 90°$, V = 38347(13) Å^3, space group Im-3).

These findings support that upon heating to 348 K in MeCN, PBA **2** degrades into separate Co- and Fe-containing products, possibly initiated by the rotation of some cyanido ligands, which are C-bound to Fe in **2** but C-bound to Co in the mononuclear product complex cation $[Co^{III}(N_2Py_2)(CN)_2]^+$. The cubic mixed-valent Fe_8 complex $[(Tp)_8Fe^{III}{}_4Fe^{II}{}_4(CN)_{12}]$ likely is a follow-up product that gradually crystallizes from the solution of initially formed fragments $\{(Tp)Fe^{II}(CN)(solv)_x\}$.

8. Van't Hoff Analysis of the LT ⇄ HT Equilibrium of 2

Reaction enthalpies and entropies for the LT ⇄ HT equilibrium of PBA **2** in MeCN neglecting the ET_1 intermediate were derived from a van't Hoff plot. Molar fractions of the LT phase x_{LT} required for calculating temperature dependent equilibrium constants

$$K = \frac{[HT]}{[LT]} = \frac{1 - x_{LT}}{x_{LT}}$$

were determined from the UV/Vis spectra of Figure 3 of the main paper. Thermodynamic constants were calculated by fitting the data in a van't Hoff plot (Figure S12) using ΔH and ΔS as fitting parameters:

$$\ln(K) = -\frac{\Delta H}{RT} + \frac{\Delta S}{R}$$

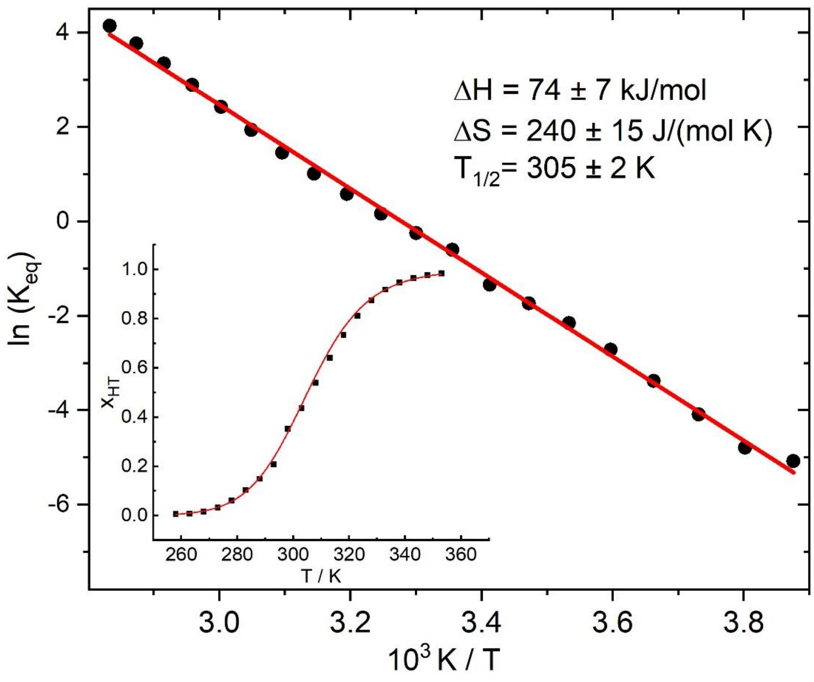

Figure S12: Van't Hoff plot (K = [HT]/[LT]) from the temperature dependent UV/Vis spectra of Figure 3 of the main paper.

9. IR Spectrum of ET_1

The IR spectrum of ET_1 in the region of the CN stretching modes was constructed from the pump-probe difference spectrum at a time delay of 22 ps (ΔA_{22}) and the inverted FTIR spectrum of the LT species at 298 K (see Figure S13). According to our kinetic analysis corresponds ΔA_{22} to the relaxed ET_1 spectrum superimposed by the bleach of the LT species. The latter is estimated by scaling the inverted FTIR spectrum of LT to the maximum of the bleach component in the 22 ps transient. Subtraction of the scaled FTIR spectrum from the 22 ps transient gives the pure absorption spectrum of ET_1.

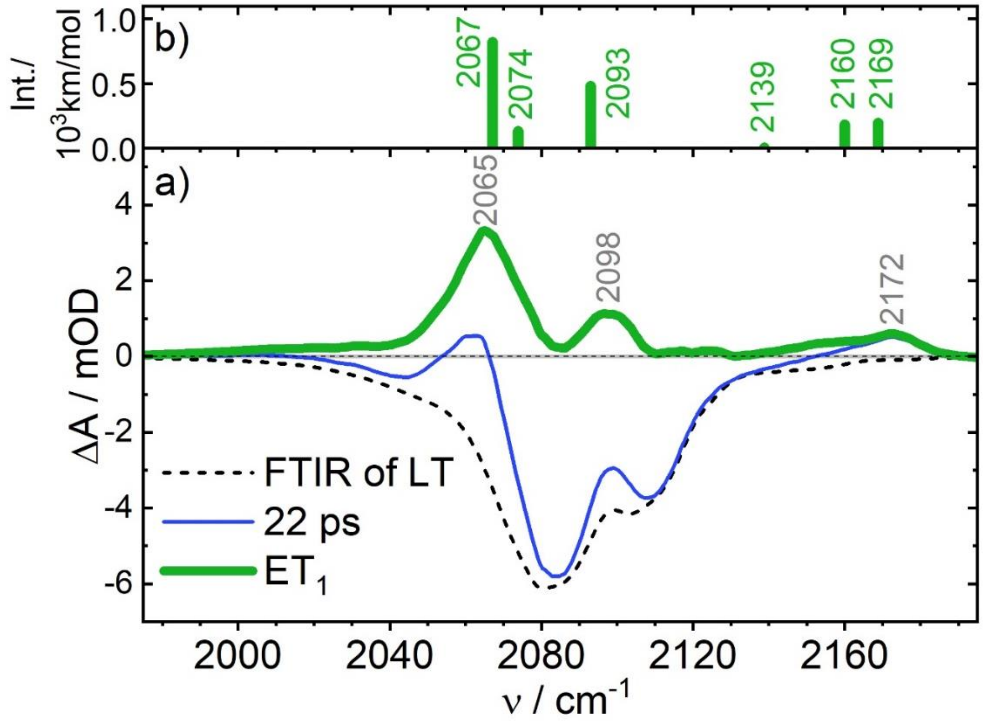

Figure S13. (a) Comparison of the transient difference spectrum (ΔA_{22}) at 22 ps pump-probe delay after 800 nm excitation of the LT species in MeCN at 298 K (blue) with the inverted FTIR spectrum (A_{FTIR}, black dashed line) that is scaled to the maximal bleach of ΔA_{22}. The spectrum of ET_1 (green) was calculated from the difference (ΔA_{22} - A_{FTIR}); (b) theoretical spectrum of ET_1.

10. UV/Vis Transients and Time Traces with Exponential Fits

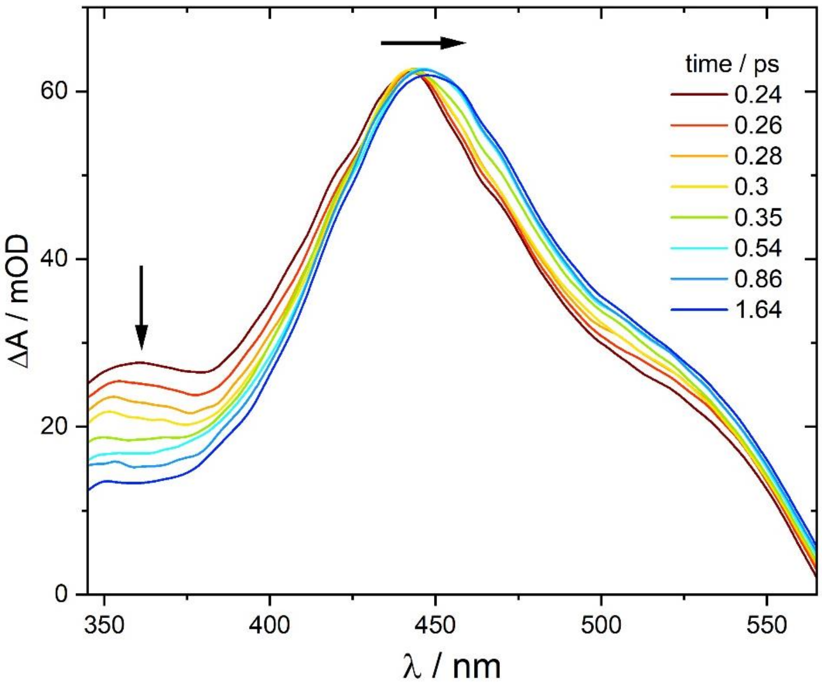

Figure S14. Early time behavior of the UV/Vis excited state absorption of PBA **2** in MeCN after 800 nm excitation showing a red-shift of the main band at 450 nm and decay of the 370 nm shoulder on a sub-picosecond timescale.

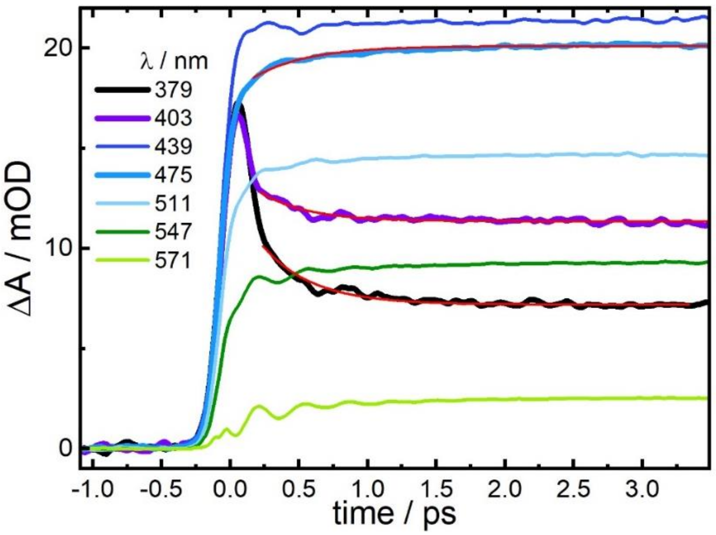

Figure S15. UV/Vis time traces for PBA **2** in MeCN after 800 nm excitation showing expanded views of transients in Fig. 5b of the main paper (red lines: exponential fits with time constants of 360±40 fs).

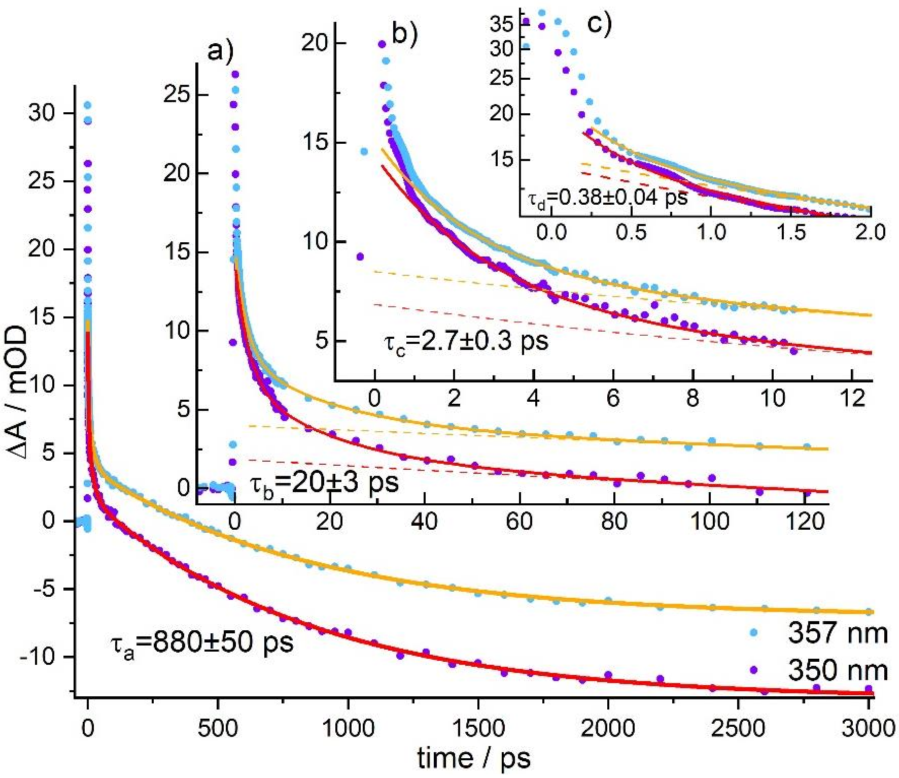

Figure S16. UV/Vis time traces at 350 and 357 nm with multi-exponential fits (red and yellow lines). Inserts (a), (b), and (c) show increasingly expanded views of the dynamics where dashed lines represent extrapolations omitting the respective exponential component shown in the panel, e.g., panel (b) illustrates the significance of the τ_c = 2.7 ps component by comparing bi-exponential fits to time points > 10 ps (including only τ_a and τ_b, dashed lines) with tri-exponential fits to time points >1 ps, adding the τ_c component and leaving τ_a and τ_b unchanged (solid lines).

11. Determining the Rate Constants of the LT ⇄ ET_1 ⇄ HT Equilibrium

The coupled equilibria

$$LT \underset{k_{-1}}{\overset{k_1}{\rightleftharpoons}} ET_1 \underset{k_{-2}}{\overset{k_2}{\rightleftharpoons}} HT \tag{S1}$$

with forward and backward rate constants k_1, k_2 and k_{-1}, k_{-2}, respectively, are defined by the equilibrium constants

$$K_1 = x_{ET1}/x_{LT} = k_1/k_{-1} \tag{S2}$$

and

$$K_2 = x_{HT}/x_{ET1} = k_2/k_{-2} \tag{S3}$$

Where the x_i denote molar fractions of the species LT, ET_1, and HT. Including mass conservation

$$x_{LT} + x_{ET1} + x_{HT} = 1 \tag{S4}$$

the molar fractions at equilibrium can be expressed in terms of the equilibrium constants

$$x_{LT} = \frac{1}{K_1 + K_1 K_2 + 1} \tag{S5}$$

$$x_{HT} = \frac{K_1 K_2}{K_1 + K_1 K_2 + 1} \tag{S6}$$

$$x_{ET1} = \frac{K_1}{K_1 + K_1 K_2 + 1} \tag{S7}$$

When the equilibrium is disturbed by an external stimulus (e.g. a light pulse transferring population from LT to ET_1), the system relaxes back to equilibrium at a rate that depends on the coefficients k_1, k_{-1}, k_2, and k_{-2}. To derive the relaxation time constants of this process the determinant (matrix) method was utilized.[6] The kinetic equations of the coupled equilibria expressed in matrix form are

$$\frac{d}{dt}\begin{pmatrix} x_{ET1} \\ x_{LT} \\ x_{HT} \end{pmatrix} = \begin{pmatrix} -k_2 - k_{-1} & k_1 & k_{-2} \\ k_{-1} & -k_1 & 0 \\ k_2 & 0 & -k_{-2} \end{pmatrix} \begin{pmatrix} x_{ET1} \\ x_{LT} \\ x_{HT} \end{pmatrix} \qquad \text{(S8)}$$

The matrix of rate constants in eq. (S8) provides three eigenvalues λ_j, which are related to the relaxation time constants by $\tau_j = -1/\lambda_j$. Two of these are given by

$$\frac{1}{\tau_{1,2}} = \frac{1}{2}(k_1 + k_{-1} + k_2 + k_{-2}) \\ \pm \frac{1}{2}\sqrt{k_1^2 + k_{-1}^2 + k_2^2 + k_{-2}^2 + 2k_1k_{-1} + 2k_2k_{-2} + 2k_2k_{-1} - 2k_1k_2 - 2k_1k_{-2} - 2k_{-1}k_{-2}} \qquad \text{(S9)}$$

The third eigenvalue $\lambda_3 = 0$ is irrelevant as after disturbance the system relaxes back to the initial state, i.e. deviations of molar fractions from equilibrium values follow double exponential decays of the form

$$\Delta x_i(t) = a_1 \exp(-t/\tau_1) + a_2 \exp(-t/\tau_2) \qquad \text{(S10)}$$

without offset. Eq. (S10) justifies fitting all UV/Vis time traces by bi-exponential decays at pump-probe delays >60 ps. Although the complete relaxation towards equilibrium could not be resolved due to a limited time window of 4 ns the absence of an offset allows to estimate the longer time constant τ_2 (10s of ns) reasonably well.

Four conditional equations are needed to determine the four unknown rate constants k_1, k_{-1}, k_2, and k_{-2}. Two are immediately given by eqs. (S9). A third equation considers the known molar fraction of LT at equilibrium taken from Figure 3 (inserting eqs. (S2) and (S3) into (S5))

$$x_{LT} = \frac{1}{\frac{k_1}{k_{-1}} + \frac{k_1k_2}{k_{-1}k_{-2}} + 1} \qquad \text{(S11)}$$

And the fourth boundary is given by the relative amplitudes of the exponential components in the recovery of the UV/Vis transients at >720 nm. Since only LT absorbs in this spectral window, the change in absorbance follows the kinetics of the low temperature species, i.e. $\Delta A_{720}(t) \propto \Delta x_{LT}(t)$, where the relative amplitudes $a_1/(a_1 + a_2)$ and $a_2/(a_1 + a_2)$ in Eq. (S10) depend on the rate constants to be determined.

12. Fs-UV/Vis (TR-UV/vis) and -IR (TR-IR) Pump Probe Spectroscopy

A 1 kHz Ti:sapphire oscillator/regenerative amplifier system (Solstice Ace, Spectra Physics) producing 4mJ/35 fs pulses at 800 nm was used to perform transient UV/Vis pump-probe experiments. A fraction of ~2 μJ focused to a diameter of about 200 μm was used to excite the sample. The probe pulse consisting of a white-light continuum was generated by focusing ~1% of the 800 nm light in a 4 mm thick sapphire crystal. About 30% of white light served as a reference, the other 70% was overlapped with the pump pulse at the sample. Every 2nd pump pulse was blocked with a synchronized chopper to measure difference spectra induced by the pump pulse. The relative polarization angle between pump and probe pulse was adjusted to 54.7° to avoid signal contributions arising from anisotropic distribution of excited molecules. Spectra were recorded in wavelength range 350-730 nm by means of two spectrographs each equipped with a 256 element linear diode array. Time delays between pump and probe pulses up to 4 ns were adjusted with a motorized translation stage (Newport, Model DL325) using retro reflectors in a quadruple pass arrangement. Experiments were performed with a quartz glass cell of 2 mm optical path length. Accumulation of photoproducts in the pump laser focus was avoided by use of a magnetic stirrer. Data acquisition was controlled with a LabVIEW program. The recorded spectra were corrected with respect to time shifts introduced by group delay dispersion within the white light probe continuum.

Transient IR absorption spectroscopy was performed with an analogous 1kHz Ti:sapphire oscillator/regenerative amplifier system (Coherent, Libra) producing 100 fs pulses at 800 nm. A home build two stage optical parametric amplifier pumped by 0.5 mJ of the regenerative amplifier output was used to produce tunable IR probe pulses with a bandwidth of about 200 cm^{-1}. The IR light was split into a reference and a probe beam. The latter passed a motorized translation stage, which allowed adjusting the pump probe time delay up to 1.8 ns. Using an extra pair of movable mirrors the path length of the 800 nm pulse could be shortened with respect to the IR pulse enabling an extra time delay of 8.7 ns. The pump energy was typically 2μJ focused to a diameter of 200 μm at the sample.

The sample cell consisted of stainless steel equipped with CaF_2 windows and a magnetic stirrer.[7] After passing the sample cell reference and probe IR pulses were dispersed in a spectrograph and recorded with a liquid nitrogen cooled HgCdTe-detector (Infrared Associates Inc.) consisting of 2 linear arrays of 32 pixel. The mid IR beam path was purged with dry nitrogen to minimize pulse distortions in air caused by CO_2 and water absorptions.

13. Computational Details

All geometries and wavefunctions were obtained with the Gaussian16 (revision C01) package,[8] using the LC-BP86 density functional[9] in combination with the def2-TZVP full electron basis set.[10] For each electronic state, the initial guess (starting molecular orbitals in the first self-consistent field iteration) was built from the fragment guess orbitals corresponding to the “ideal” electronic. Normal-mode (frequency) analyses were computed to confirm minima on the potential energy surface. The frequencies were scaled by a factor of 0.901, calculated so as to overlap the highest CN computed stretch frequency of the LT state with the experimentally observed 2079 cm^{-1} band. These frequencies were used to calculate the CN vibrational shifts provided in the manuscript.

All chemical bonding indicators (effective fragment orbitals and effective oxidation states) used to confirm/characterize the electronic configurations of the resulting wavefunctions were evaluated with the APOST-3D software,[11] using the topological fuzzy Voronoi cells (TFVC)[12] real-space atomic definition and the default integration setup for the numerical integrations.

14. Raman Active Mode Causing the Wavepacket in the UV/Vis Transients

In the UV/Vis transients of Figure 6b a ~108 cm^{-1} coherent oscillation is observed, which we attribute to a wavepacket excited by impulsive stimulated Raman scattering (ISRS) in the ground state of LT. Our DFT calculations indeed predict a Raman active mode for LT at 106.5 cm^{-1} (see Figure S17) corresponding to the totally symmetric breathing mode of the Fe_2Co_2 square including all four capping ligands of the LT species. An animated gif visualizing this mode is provided as a separate file. The photo-induced changes in bond lengths between metal centers and ligands effectively couple this mode to the IVCT transition. These changes are illustrated in Figure S18 showing equilibrium structures of LT and ET_1 (we consider the structure of ET_1 being very similar to the low-spin species [$Fe^{II}_{LS}Co^{III}_{LS}Fe^{III}_{LS}Co^{II}_{LS}$] which is initially populated by the 800 nm excitation of the LT species).

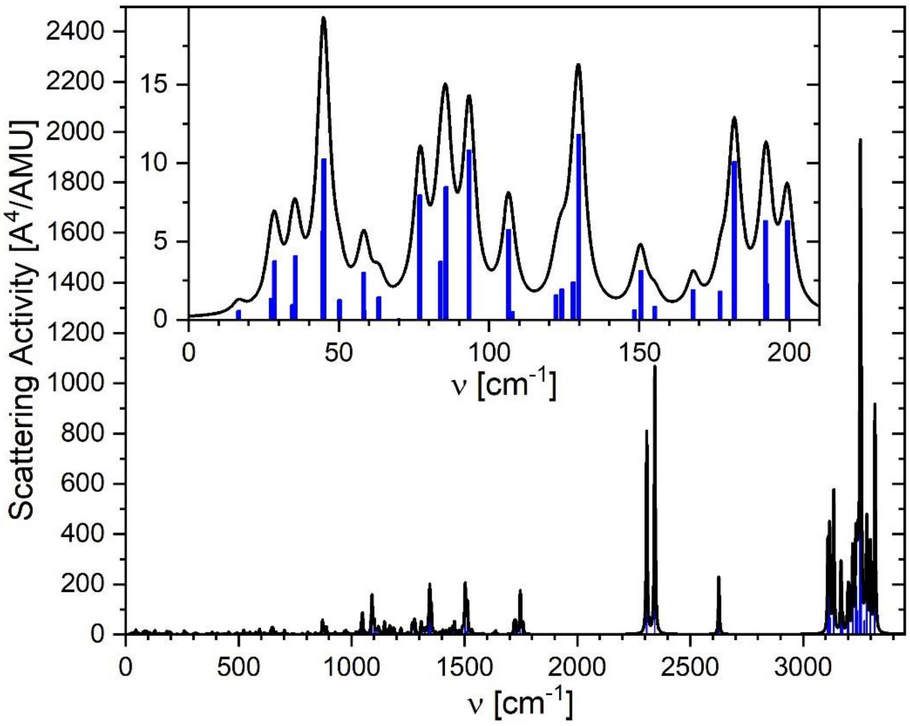

Figure S17. Calculated Raman scattering activity (blue bars) of the LT species in the ground state (black line: convolution of the stick spectrum with a Lorentzian line shape function of 5 cm^{-1} width (FWHM)). The insert enlarges the low frequency spectrum. Note, that this spectrum does not take into account resonance enhancement when the excitation is in resonance with an electronic transition (e.g. the IVCT band).

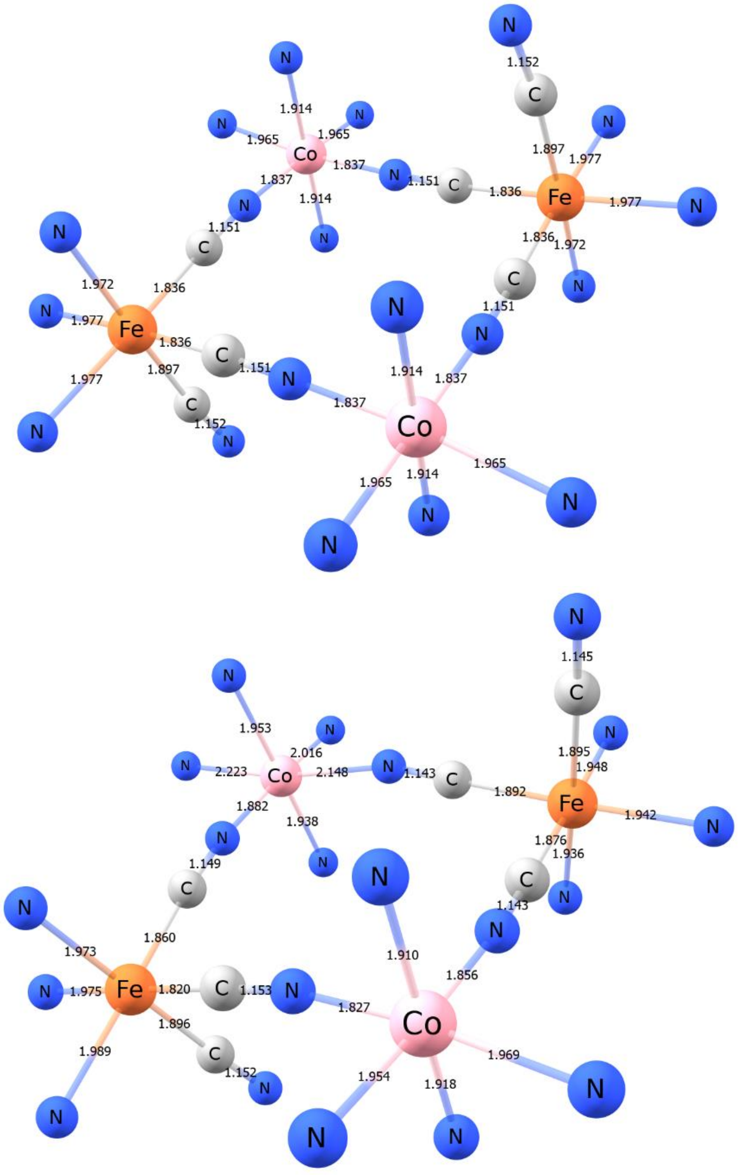

Figure S18. Central structural motif for the LT (top) and ET_1 (bottom) states, respectively, with bond lengths provided in angstroms. The remaining parts of the molecules have been omitted for clarity.

15. Mulliken-Hush Analysis of the IVCT Absorption Band

The Mulliken-Hush expression, eq. (S12),[13,14] was applied to calculate the matrix element H_{ab} coupling reactant and product electronic states of the primary electron transfer process $[Fe^{II}_{LS}Co^{III}_{LS}]_2 \rightarrow [Fe^{II}_{LS}Co^{III}_{LS}Fe^{III}_{LS}Co^{II}_{LS}]$ responsible for the near infrared IVCT absorption band of the LT species of PBA **2**.

$$H_{ab} = 2.06 \cdot 10^{-2} \frac{\left(\nu_{max}\, \varepsilon_{max}\, \Delta\nu_{1/2}\right)^{1/2}}{r_{ab}} \qquad \text{(S12)}$$

In eq. S12 ε_{max} is the intensity of the IVCT absorption band in $M^{-1}cm^{-1}$, ν_{max} and $\Delta\nu_{1/2}$ correspond to the band maximum and its width (FWHM) in wavenumber, respectively, and r_{ab} is the separation of the donor and acceptor charge centroids in Å. A Gaussian fit to the IVCT band (Figure S19) results in a matrix element of $H_{ab} = 2400\ cm^{-1}$ ($\nu_{max} = 12800\ cm^{-1}$, $\varepsilon_{max} = 5000\ M^{-1}cm^{-1}$, $\Delta\nu_{1/2} = 5300\ cm^{-1}$, $r_{ab} = 5$Å) implying moderate coupling between the low spin states $[Fe^{II}_{LS}Co^{III}_{LS}]_2$ and $[Fe^{II}_{LS}Co^{III}_{LS}Fe^{III}_{LS}Co^{II}_{LS}]$.

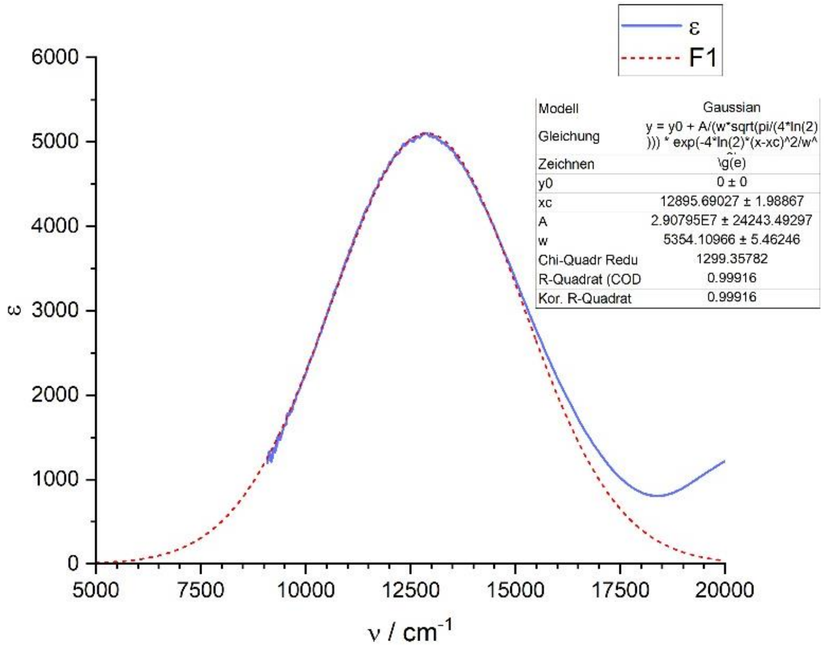

Figure S19. IVCT absorption band of the LT species fitted with a Gaussian function.

16. Cartesian Coordinates (XYZ) of the Studied Systems

192

LT state

Co	3.407235249	0.000131236	-0.000014288
Fe	-0.000115890	3.388589689	0.265428958
N	1.328955634	4.824585710	0.547261881
N	1.218789927	5.958861797	-0.129786012
N	-1.329338759	4.824399969	0.547520649
N	-1.219459866	5.958699340	-0.129537828
N	-0.000331265	3.808938462	-1.661404891
N	-0.000474143	5.064427889	-2.075404066
N	2.130568677	1.320064927	-0.040711722
N	-2.130583494	1.319797164	-0.040481530
N	0.000198971	2.550630107	3.193001544
N	4.820535916	1.345784000	-0.226405840
N	4.820553908	-1.345473373	0.226556656
N	3.359669094	0.064300857	-1.911890413
N	3.359432023	-0.064044735	1.911853370
C	2.333270023	4.964815035	1.380934070
H	2.565959313	4.176257853	2.083254410
C	2.904145380	6.220693408	1.236063860

H	3.733655060	6.640181738	1.782762697
C	2.151926800	6.819904091	0.264123478
H	2.203995196	7.809570324	-0.165441975
C	-2.333438830	4.964523987	1.381469068
H	-2.565862474	4.175936113	2.083843385
C	-2.904472412	6.220349972	1.236770841
H	-3.733878902	6.639748871	1.783694049
C	-2.152572926	6.819646911	0.264637309
H	-2.204849817	7.809315260	-0.164899039
C	-0.000437100	3.043626978	-2.730292662
H	-0.000349257	1.966486159	-2.626442156
C	-0.000655121	3.828563000	-3.874078594
H	-0.000774186	3.506317339	-4.903091271
C	-0.000670997	5.113654074	-3.402101172
H	-0.000804879	6.062213289	-3.918668127
C	1.301520440	2.115111939	0.030988620
C	-1.301609342	2.114924610	0.031201349
C	0.000064560	2.931693290	2.106083117
C	6.049051592	0.582263252	-0.461406048
H	6.044499609	0.241535547	-1.499339067
H	6.926067101	1.221304577	-0.332431794
C	6.049007141	-0.581904999	0.461731492
H	6.044295347	-0.241178352	1.499663982

H	6.926065514	-1.220912986	0.332880536
C	4.964646757	2.272747491	0.914549522
H	5.168264637	1.675658280	1.803376268
H	3.987126729	2.740781484	1.044821319
C	6.029561994	3.305967658	0.750943263
C	5.764144678	4.504747276	0.122100242
H	4.759610152	4.722984207	-0.228826826
C	6.752321806	5.448619039	-0.024135774
H	6.529581048	6.390225923	-0.513991979
C	8.014829182	5.208643507	0.465302380
H	8.792123885	5.956627081	0.354618079
C	8.284109492	4.027443685	1.115127279
H	9.271145185	3.843909669	1.524803076
C	7.293730458	3.085862202	1.259023803
H	7.505126715	2.166202415	1.799597413
C	4.964857369	-2.272417285	-0.914391827
H	5.168541926	-1.675303731	-1.803187351
H	3.987382851	-2.740512133	-1.044786923
C	4.489586348	2.089397100	-1.438550889
H	5.368056566	2.615615719	-1.822076862
H	3.726761998	2.831784096	-1.189650253
C	3.918364908	1.152182916	-2.431873736
C	3.889920573	1.401822812	-3.776171277

H	4.370499771	2.290945897	-4.167784667
C	3.223816065	0.524861809	-4.599600181
H	3.178900558	0.707411555	-5.668023335
C	2.606134450	-0.570210182	-4.050564498
H	2.025112648	-1.277282005	-4.632031868
C	2.701100599	-0.772121995	-2.700168718
H	2.199925293	-1.613578910	-2.239535269
C	4.489467812	-2.089108698	1.438653020
H	3.726715430	-2.831532207	1.189643373
H	5.367909983	-2.615286041	1.822299646
C	3.918065394	-1.151925207	2.431903370
C	3.889416267	-1.401588387	3.776193503
H	4.369947310	-2.290711471	4.167865102
C	3.223170469	-0.524652254	4.599533505
H	3.178090388	-0.707222639	5.667946604
C	2.605559764	0.570422382	4.050421620
H	2.024429480	1.277473567	4.631805909
C	2.700733879	0.772357479	2.700044361
H	2.199610962	1.613811748	2.239345294
B	-0.000439746	6.167294132	-1.026999688
H	-0.000563045	7.255935031	-1.527670158
Co	-3.407228899	-0.000161399	0.000102131
Fe	0.000130707	-3.388605564	-0.265516801

N	-1.328906950	-4.824607406	-0.547489428
N	-1.218725897	-5.958941174	0.129459510
N	1.329383210	-4.824366630	-0.547719620
N	1.219519663	-5.958726328	0.129239901
N	0.000341319	-3.809121028	1.661281063
N	0.000495839	-5.064646969	2.075170169
N	-2.130581378	-1.320121549	0.040773636
N	2.130602545	-1.319840556	0.040537094
N	-0.000204262	-2.550407323	-3.193020595
N	-4.820563963	-1.345775533	0.226536018
N	-4.820521099	1.345476019	-0.226455054
N	-3.359628877	-0.064317261	1.911976668
N	-3.359455836	0.063998696	-1.911767114
C	-2.333207580	-4.964783813	-1.381186487
H	-2.565902162	-4.176171068	-2.083442797
C	-2.904051187	-6.220691291	-1.236442751
H	-3.733540758	-6.640151575	-1.783193976
C	-2.151838428	-6.819968650	-0.264539940
H	-2.203888302	-7.809675630	0.164935023
C	2.333509211	-4.964391693	-1.381654280
H	2.565928092	-4.175738730	-2.083956629
C	2.904577189	-6.220212386	-1.237044426
H	3.734011726	-6.639540376	-1.783979805

C	2.152667119	-6.819614102	-0.264983391
H	2.204959886	-7.809317906	0.164468818
C	0.000405879	-3.043903209	2.730236040
H	0.000300044	-1.966752864	2.626478669
C	0.000606437	-3.828938716	3.873952649
H	0.000693222	-3.506784603	4.902993903
C	0.000658826	-5.113989043	3.401863042
H	0.000796941	-6.062593238	3.918347445
C	-1.301532611	-2.115164857	-0.030979624
C	1.301611458	-2.114945248	-0.031184415
C	-0.000056622	-2.931558879	-2.106132860
C	-6.049052121	-0.582218272	0.461555805
H	-6.044466800	-0.241477337	1.499484061
H	-6.926088268	-1.221238430	0.332609597
C	-6.048998145	0.581937279	-0.461597081
H	-6.044316514	0.241197932	-1.499525337
H	-6.926037467	1.220969079	-0.332735012
C	-4.964739363	-2.272747491	-0.914401353
H	-5.168307500	-1.675661984	-1.803241857
H	-3.987255848	-2.740862448	-1.044657274
C	-6.029746677	-3.305868172	-0.750770222
C	-5.764454247	-4.504633503	-0.121846766
H	-4.759950942	-4.722937110	0.229125282

C	-6.752719218	-5.448408956	0.024415709
H	-6.530076887	-6.390006315	0.514334886
C	-8.015192198	-5.208349284	-0.465071130
H	-8.792556752	-5.956257187	-0.354364074
C	-8.284350797	-4.027162163	-1.114969584
H	-9.271358973	-3.843563058	-1.524681365
C	-7.293883390	-3.085676990	-1.258892037
H	-7.505183866	-2.166028316	-1.799522270
C	-4.964782755	2.272435806	0.914486021
H	-5.168472074	1.675336540	1.803290012
H	-3.987294478	2.740506841	1.044863124
C	-4.489611219	-2.089388633	1.438680008
H	-5.368088845	-2.615575501	1.822231382
H	-3.726816503	-2.831802617	1.189768259
C	-3.918338449	-1.152182387	2.431981159
C	-3.889875593	-1.401808525	3.776281346
H	-4.370466962	-2.290916792	4.167912728
C	-3.223740922	-0.524850696	4.599688554
H	-3.178809540	-0.707389859	5.668113295
C	-2.606049782	0.570205419	4.050631174
H	-2.025004695	1.277273538	4.632079493
C	-2.701036040	0.772104532	2.700235924
H	-2.199850150	1.613545571	2.239585012

C	-4.489441354	2.089091235	-1.438565176
H	-3.726667804	2.831500457	-1.189577226
H	-5.367879291	2.615283396	-1.822201219
C	-3.918074919	1.151886577	-2.431816585
C	-3.889436376	1.401540761	-3.776108834
H	-4.369956835	2.290669666	-4.167780963
C	-3.223213332	0.524588753	-4.599450953
H	-3.178141189	0.707152258	-5.667865640
C	-2.605613740	-0.570492234	-4.050339068
H	-2.024501448	-1.277557177	-4.631724416
C	-2.700777801	-0.772418334	-2.699959693
H	-2.199664409	-1.613878424	-2.239261155
B	0.000495839	-6.167423780	1.026670539
H	0.000638188	-7.256107543	1.527247875
C	-6.029726568	3.305611521	0.750778689
H	-7.505341031	2.165769019	1.799276731
C	-8.284344447	4.026979597	1.114746272
C	-7.293935250	3.085443623	1.258732755
H	-9.271411890	3.843396367	1.524323641
C	-8.015055670	5.208196352	0.464956298
H	-8.792374186	5.956145001	0.354200558
C	-6.752510722	5.448235386	-0.024354325
H	-6.529765201	6.389857087	-0.514180366

C	-5.764303431	4.504409661	0.121972181
H	-4.759743505	4.722698451	-0.228849051
C	6.029823937	-3.305569187	-0.750680262
H	4.759862041	-4.722691572	0.228923136
C	6.752647250	-5.448182468	0.024445343
C	5.764420909	-4.504377381	-0.121885396
H	6.529917604	-6.389813165	0.514261330
C	8.015192198	-5.208111684	-0.464849404
H	8.792526589	-5.956042870	-0.354091019
C	8.284461924	-4.026883816	-1.114627736
H	9.271529368	-3.843275186	-1.524192405
C	7.294032618	-3.085369538	-1.258618982
H	7.505424641	-2.165685938	-1.799154491

192

ET1 state

Co	3.423488929	0.242284333	0.147588064
Fe	0.327788263	-3.336352486	-0.493339778
N	1.783275470	-4.544223370	-1.115695616
N	1.860052209	-5.776452282	-0.629846341
N	-0.859505563	-4.867483464	-0.865340801
N	-0.552871690	-6.066096921	-0.388116586
N	0.574756344	-4.034768028	1.336126515

N	0.784415312	-5.323655419	1.540600604
N	2.127035361	-1.040936629	0.072569251
N	-1.993773713	-1.595586883	0.276396156
N	0.225865021	-2.107503958	-3.274344025
N	4.745126042	-1.167317915	0.436683945
N	4.908886291	1.530169462	0.040036492
N	3.267268925	0.157024353	2.050168661
N	3.508332446	0.336587952	-1.765793570
C	2.648715227	-4.464099055	-2.102105291
H	2.712255126	-3.560968649	-2.693151299
C	3.315410296	-5.669065285	-2.256869528
H	4.076787347	-5.920217570	-2.977680380
C	2.769115751	-6.478269376	-1.300526116
H	2.949950434	-7.515513935	-1.059046662
C	-1.987965463	-4.986312331	-1.523108650
H	-2.410605044	-4.129569074	-2.028938070
C	-2.437715853	-6.297484435	-1.468504967
H	-3.326019771	-6.713139406	-1.916838220
C	-1.484392984	-6.948667732	-0.736375542
H	-1.396610946	-7.985359831	-0.446441444
C	0.615815207	-3.440637976	2.507940849
H	0.447257436	-2.374469646	2.577484794
C	0.863404541	-4.373920373	3.504131308

H	0.935550451	-4.211518521	4.567759057
C	0.960745638	-5.564142666	2.834126234
H	1.135081495	-6.569101063	3.189371918
C	1.426974078	-1.937006223	-0.118964867
C	-1.109193085	-2.287461794	0.021594136
C	0.200184049	-2.628115944	-2.246866491
C	5.990803465	-0.471819198	0.774780581
H	5.932702449	-0.154831971	1.818618160
H	6.840049339	-1.154148810	0.690029670
C	6.115302996	0.710188677	-0.116940234
H	6.175303759	0.391855224	-1.160055486
H	7.008512917	1.303428124	0.094489890
C	4.928213432	-2.083824863	-0.711105741
H	5.288580493	-1.492379328	-1.553349543
H	3.935191157	-2.459613737	-0.962688247
C	5.857039627	-3.221677660	-0.449881626
C	5.378389810	-4.408595770	0.065576174
H	4.313155478	-4.531482898	0.246695025
C	6.234325543	-5.454710398	0.310956193
H	5.845794082	-6.385527888	0.709096984
C	7.575098764	-5.330999870	0.031012433
H	8.248088926	-6.160361381	0.218770342
C	8.057568183	-4.163772445	-0.512076357

H	9.108720579	-4.072743375	-0.762087207
C	7.199240039	-3.118365856	-0.754335289
H	7.580941939	-2.211139089	-1.216992841
C	5.018373064	2.438909677	1.198448883
H	5.144435785	1.825467301	2.090247488
H	4.052039844	2.939653174	1.283730030
C	4.300396486	-1.922621598	1.608250095
H	5.131510631	-2.491064328	2.033740353
H	3.526610757	-2.630512058	1.300857910
C	3.715101577	-0.975395910	2.580307425
C	3.591953035	-1.241247151	3.917210244
H	3.975368940	-2.173782350	4.315099675
C	2.967944048	-0.319045197	4.721949666
H	2.855967496	-0.508423446	5.784264526
C	2.486084241	0.839407940	4.163602050
H	1.970002541	1.601006187	4.736992587
C	2.653368282	1.042234701	2.821983204
H	2.262284599	1.940051638	2.361895866
C	4.701057748	2.296404360	-1.184462728
H	3.956133347	3.071685543	-0.983797128
H	5.625684392	2.790346342	-1.495450672
C	4.168310669	1.393763443	-2.230334993
C	4.266362451	1.650115424	-3.568935941

H	4.832106377	2.508699689	-3.912314825
C	3.617079958	0.818886976	-4.453407029
H	3.670515748	1.008537223	-5.520235771
C	2.889062241	-0.235228812	-3.965646896
H	2.307721874	-0.900482403	-4.595005335
C	2.864726438	-0.451972405	-2.613374653
H	2.273315828	-1.263426557	-2.209085352
B	0.775384373	-6.256071024	0.335650250
H	0.948909001	-7.397033795	0.659432112
Co	-3.380975887	-0.150663642	0.344755803
Fe	-0.052329809	3.657063533	0.111475421
N	-1.458023024	4.991953361	0.291840194
N	-1.544183134	5.973431105	-0.595880041
N	1.173039379	5.142310080	-0.147324534
N	0.856782417	6.103761640	-1.004862087
N	-0.390337014	3.596666417	-1.789801813
N	-0.561186122	4.726294740	-2.465871258
N	-2.106491642	1.465845322	0.509991399
N	2.153264031	1.594818518	0.041092201
N	0.669865371	3.526264676	3.056876518
N	-5.091792174	1.169645236	-0.037631382
N	-4.951861834	-1.638177714	0.488542257
N	-3.311507614	0.122847440	-1.753506604

N	-3.607302879	-0.354742436	2.426279274
C	-2.413187958	5.166630537	1.177997241
H	-2.513936427	4.487296069	2.012809277
C	-3.146950423	6.294454896	0.855818256
H	-3.985501626	6.711459797	1.390416397
C	-2.553114594	6.775258994	-0.280039542
H	-2.776136346	7.642746667	-0.883978953
C	2.316536909	5.471412820	0.417470578
H	2.732815779	4.853309451	1.200759800
C	2.762485160	6.672688568	-0.096304968
H	3.649530166	7.222388369	0.175647688
C	1.794678710	7.037555752	-0.997468422
H	1.706523072	7.911245382	-1.626819983
C	-0.493620242	2.595263033	-2.644402961
H	-0.400270729	1.569185701	-2.310959910
C	-0.734766844	3.091856193	-3.908120037
H	-0.870298133	2.532517428	-4.819510900
C	-0.768279639	4.454360077	-3.743563375
H	-0.927298990	5.250891431	-4.456139700
C	-1.303439237	2.268061627	0.385112447
C	1.302192495	2.357009972	0.020734223
C	0.340922443	3.633557479	1.965433096
C	-6.200526992	0.273230089	-0.321572547

H	-6.087382017	-0.080740277	-1.349469720
H	-7.158001256	0.804560031	-0.276322072
C	-6.192137945	-0.895098025	0.616729096
H	-6.268070118	-0.540553501	1.648125717
H	-7.061094614	-1.540104236	0.443902452
C	-5.339715420	2.031759114	1.117980074
H	-5.517007261	1.390872018	1.983204455
H	-4.407254306	2.571189701	1.308054191
C	-6.473358082	2.993817111	0.965953800
C	-6.277095765	4.236037419	0.398879523
H	-5.279402972	4.534299180	0.087167135
C	-7.326200245	5.110382171	0.245470509
H	-7.158278016	6.085380140	-0.199280743
C	-8.586949694	4.755277778	0.664953548
H	-9.414427334	5.446281134	0.548270493
C	-8.791453417	3.527441566	1.247549652
H	-9.779613082	3.249091687	1.596897126
C	-7.738676447	2.656264469	1.398430786
H	-7.902418176	1.695918360	1.880883272
C	-4.926801058	-2.513989345	-0.684754302
H	-5.093630007	-1.895174762	-1.568499887
H	-3.907072266	-2.900346946	-0.754049004
C	-4.749346230	1.948205201	-1.206932653

H	-5.627589431	2.438186291	-1.643945746
H	-4.058844534	2.738227146	-0.892568558
C	-4.048055139	1.115625764	-2.224956436
C	-4.111840048	1.393787256	-3.566408062
H	-4.746423584	2.198156783	-3.921595535
C	-3.360785658	0.639319143	-4.437050689
H	-3.400187138	0.837338328	-5.503420106
C	-2.565651332	-0.362739891	-3.940358044
H	-1.926765056	-0.977673489	-4.564400898
C	-2.580947730	-0.594194612	-2.588259901
H	-1.968060461	-1.377943689	-2.157264082
C	-4.654838349	-2.374400853	1.696137440
H	-3.811775910	-3.039215757	1.480873427
H	-5.497669537	-2.998712001	2.015602274
C	-4.231108133	-1.460519153	2.793682805
C	-4.428745252	-1.769958724	4.117775829
H	-4.956590033	-2.678087210	4.386465048
C	-3.950448925	-0.914237308	5.077050354
H	-4.098274589	-1.134216819	6.129047846
C	-3.285846751	0.224944782	4.688648012
H	-2.891192180	0.925818881	5.413906070
C	-3.136371641	0.464962648	3.349109891
H	-2.614569535	1.345077019	2.983801373

B	-0.502204555	6.048277406	-1.706345269
H	-0.671364001	6.987290263	-2.426324783
C	-5.908049139	-3.639931949	-0.658610829
H	-7.442940450	-2.552068411	-1.673018215
C	-8.079911634	-4.519452583	-1.155323054
C	-7.170476610	-3.489313816	-1.194146143
H	-9.066832495	-4.388100206	-1.585200192
C	-7.730996498	-5.720314514	-0.584551944
H	-8.444451257	-6.536496865	-0.556965934
C	-6.468123989	-5.889668697	-0.066968439
H	-6.181258590	-6.842865092	0.363817826
C	-5.562365689	-4.856736403	-0.107965388
H	-4.555223377	-5.008286417	0.271350451
C	6.130434290	3.432325659	1.119474470
H	5.020596138	4.882958194	-0.000130178
C	7.006137969	5.538187580	0.400101922
C	5.972588655	4.635050824	0.461417690
H	6.870713574	6.481448143	-0.117774218
C	8.207951361	5.254304095	1.005716179
H	9.020998866	5.970435433	0.962458055
C	8.369688037	4.068979337	1.681957606
H	9.307636719	3.849765545	2.179856775
C	7.333945812	3.167316868	1.739383392

H	7.459767228	2.243987237	2.299688434

192

HT state

Co	3.443995605	-0.000789003	0.000187329
Fe	-0.000921827	-3.695394487	-0.383954078
N	1.341650597	-5.047169832	-0.686815976
N	1.222305781	-6.229927022	-0.095597987
N	-1.344177947	-5.046373949	-0.687263660
N	-1.225627956	-6.229240679	-0.096102293
N	-0.001568481	-4.165731988	1.521768244
N	-0.001875404	-5.441283584	1.877821452
N	2.125454179	-1.565216872	-0.011840341
N	-2.126126234	-1.564008231	-0.012462653
N	-0.000230721	-2.667578808	-3.235403988
N	5.084353000	-1.376029655	0.383958841
N	5.084533979	1.374237861	-0.384284285
N	3.477348060	-0.073060328	2.106328655
N	3.476459042	0.071742676	-2.105900551
C	2.423781558	-5.081627208	-1.437620066
H	2.692649638	-4.226635527	-2.042181260
C	3.032177170	-6.314793823	-1.322181107
H	3.928816159	-6.658759035	-1.812356935

C	2.225584569	-7.013234765	-0.459821162
H	2.295785223	-8.025343405	-0.088423402
C	-2.426093533	-5.080203722	-1.438412244
H	-2.694319193	-4.225018891	-2.042987726
C	-3.035174959	-6.313052830	-1.323241578
H	-3.931848874	-6.656520086	-1.813702632
C	-2.229222134	-7.011979028	-0.460670492
H	-2.300077909	-8.024074967	-0.089362691
C	-0.001943668	-3.442192169	2.620257132
H	-0.001756339	-2.362174213	2.564434224
C	-0.002516238	-4.275076413	3.725069160
H	-0.002875020	-3.994310840	4.765803113
C	-0.002465437	-5.539843905	3.200077173
H	-0.002810990	-6.507894835	3.679427622
C	1.328476200	-2.377001760	-0.120387295
C	-1.329554134	-2.376204819	-0.120880488
C	-0.000496897	-3.089038324	-2.170158544
C	6.264563260	-0.539800482	0.519371595
H	6.243925348	-0.091677313	1.516274855
H	7.180826258	-1.138266084	0.463599488
C	6.264374873	0.537621330	-0.520511442
H	6.242879693	0.089490752	-1.517394065
H	7.180881293	1.135775775	-0.465382286

C	5.197348218	-2.345635307	-0.707685140
H	5.354306953	-1.790560124	-1.634389334
H	4.221541667	-2.830857507	-0.790325692
C	6.270920267	-3.371446463	-0.542769696
C	6.018202675	-4.555633490	0.118338321
H	5.018452441	-4.760329304	0.491917350
C	7.010825950	-5.491606224	0.284421659
H	6.798345410	-6.419872020	0.803627092
C	8.269678829	-5.257392373	-0.217405064
H	9.052384369	-5.997084799	-0.090592499
C	8.528298334	-4.090156481	-0.895670595
H	9.513633251	-3.909496427	-1.310946674
C	7.532140155	-3.156973041	-1.058575165
H	7.736349126	-2.247316291	-1.617814973
C	5.198502354	2.343688993	0.707388271
H	5.355648947	1.788484161	1.633981338
H	4.222952983	2.829336651	0.790533130
C	4.771436734	-2.028804717	1.635414350
H	5.646581602	-2.530047758	2.065524856
H	4.022286853	-2.800702870	1.432091222
C	4.172189009	-1.077587974	2.614620866
C	4.270210628	-1.275025064	3.969301394
H	4.864946588	-2.096371127	4.353303627

C	3.599287961	-0.425282821	4.814762063
H	3.660671463	-0.562563571	5.889230009
C	2.847984328	0.593255322	4.281691128
H	2.277818597	1.278494350	4.897752402
C	2.820801551	0.736915303	2.919673558
H	2.229717973	1.522294776	2.460282617
C	4.771111820	2.027262695	-1.635482085
H	4.022370463	2.799417499	-1.431635071
H	5.646187894	2.528234796	-2.066052445
C	4.170959730	1.076380392	-2.614459996
C	4.267870077	1.274221244	-3.969156928
H	4.862360499	2.095636100	-4.353391470
C	3.596175869	0.424813441	-4.814347718
H	3.656684642	0.562461440	-5.888819367
C	2.845262770	-0.593848001	-4.280983089
H	2.274463084	-1.278879591	-4.896690873
C	2.819230424	-0.737931324	-2.918978219
H	2.228512507	-1.523465316	-2.459395186
B	-0.001901334	-6.497855284	0.780069708
H	-0.002308800	-7.608876148	1.221657010
Co	-3.443974438	0.000843509	-0.000095252
Fe	0.000820225	3.695478626	0.384085843
N	-1.341731561	5.047285722	0.686757767

N	-1.222299431	6.230015924	0.095499560
N	1.344102275	5.046458618	0.687386959
N	1.225635364	6.229281955	0.096127693
N	0.001538318	4.165680658	-1.521660821
N	0.001962718	5.441208441	-1.877801343
N	-2.125583828	1.565350753	0.012009678
N	2.126007699	1.563973834	0.012908750
N	-0.000023284	2.667979924	3.235662227
N	-5.084316487	1.375984675	-0.384125002
N	-5.084518633	-1.374224631	0.384337732
N	-3.477088763	0.072928563	-2.106264625
N	-3.476628379	-0.071592390	2.106025966
C	-2.423874693	5.081841525	1.437546510
H	-2.692819504	4.226881595	2.042118288
C	-3.032182991	6.315042536	1.322057280
H	-3.928806105	6.659088183	1.812205590
C	-2.225533239	7.013397752	0.459676167
H	-2.295670921	8.025494221	0.088232369
C	2.426015744	5.080277806	1.438533426
H	2.694165731	4.225139543	2.043207334
C	3.035193480	6.313071351	1.323246870
H	3.931892266	6.656512148	1.813680936
C	2.229280343	7.011986436	0.460631862

H	2.300197503	8.024050096	0.089247331
C	0.001780152	3.442067283	-2.620100496
H	0.001487517	2.362053031	-2.564204562
C	0.002387119	4.274875854	-3.724970203
H	0.002678695	3.994039372	-4.765685107
C	0.002489250	5.539678273	-3.200064473
H	0.002885604	6.507696393	-3.679481069
C	-1.328581506	2.377109183	0.120563511
C	1.329445123	2.376191589	0.121236624
C	0.000316448	3.089292858	2.170359102
C	-6.264522514	0.539745448	-0.519520293
H	-6.243847558	0.091550310	-1.516390216
H	-7.180787099	1.138214753	-0.463821742
C	-6.264365348	-0.537597517	0.520447412
H	-6.242892923	-0.089380154	1.517291405
H	-7.180873355	-1.135752492	0.465346831
C	-5.197381027	2.345694575	0.707416847
H	-5.354430781	1.790709352	1.634160200
H	-4.221570243	2.830899841	0.790092854
C	-6.270902275	3.371520019	0.542296611
C	-6.018073556	4.555636136	-0.118895544
H	-5.018277283	4.760258923	-0.492394668
C	-7.010644972	5.491631095	-0.285159332

H	-6.798078704	6.419841328	-0.804428795
C	-8.269555531	5.257510909	0.216567377
H	-9.052220324	5.997221856	0.089610875
C	-8.528287750	4.090345398	0.894911225
H	-9.513670293	3.909761015	1.310107399
C	-7.532181431	3.157137086	1.057994128
H	-7.736479304	2.247534312	1.617289500
C	-5.198409748	-2.343739265	-0.707282436
H	-5.355540994	-1.788604814	-1.633921012
H	-4.222837622	-2.829355701	-0.790353739
C	-4.771323491	2.028636968	-1.635623375
H	-5.646435020	2.529858312	-2.065828074
H	-4.022163555	2.800532475	-1.432326177
C	-4.172051952	1.077300102	-2.614698655
C	-4.270185756	1.274477366	-3.969410404
H	-4.865018556	2.095701718	-4.353524294
C	-3.599239806	0.424638283	-4.814752538
H	-3.660695276	0.561705774	-5.889243238
C	-2.847798587	-0.593727877	-4.281540842
H	-2.277605339	-1.278998656	-4.897541260
C	-2.820513679	-0.737137029	-2.919504221
H	-2.229304156	-1.522346106	-2.459993157
C	-4.771189609	-2.027185964	1.635587921

H	-4.022462540	-2.799377810	1.431833513
H	-5.646309605	-2.528111498	2.066126001
C	-4.171084087	-1.076267677	2.614564244
C	-4.268014542	-1.274119113	3.969262764
H	-4.862467392	-2.095571011	4.353476668
C	-3.596393890	-0.424674797	4.814470487
H	-3.656912717	-0.562331791	5.888940019
C	-2.845532650	0.594039563	4.281125967
H	-2.274791174	1.279104492	4.896851213
C	-2.819477021	0.738128707	2.919126388
H	-2.228791912	1.523694979	2.459559231
B	0.001963777	6.497858459	-0.780127388
H	0.002410402	7.608848101	-1.221792479
C	-6.272308828	-3.369133958	-0.541893907
H	-7.737791134	-2.244420104	-1.616257605
C	-8.530125054	-4.086963955	-0.893779315
C	-7.533679531	-3.154165226	-1.057124161
H	-9.515576390	-3.905919189	-1.308610886
C	-8.271653189	-5.254305682	-0.215637083
H	-9.054588392	-5.993694889	-0.088475790
C	-7.012666958	-5.489009022	0.285623420
H	-6.800315537	-6.417360016	0.804729897
C	-6.019755810	-4.553419941	0.119098748

H	-5.019914558	-4.758496763	0.492227977
C	6.272446943	3.369047702	0.542044194
H	5.020141045	4.758511580	-0.492053877
C	7.012924667	5.488912712	-0.285395874
C	6.019964306	4.553367553	-0.118917241
H	6.800627751	6.417290164	-0.804476950
C	8.271888673	5.254134229	0.215884738
H	9.054861447	5.993490627	0.088764192
C	8.530289099	4.086761809	0.894001040
H	9.515723501	3.905657246	1.308846899
C	7.533794363	3.154008060	1.057299319
H	7.737849873	2.244233834	1.616405774

17. References

[1] J. Kim, S. Han, I.-K. Cho, K. Y. Choi, M. Heu, S. Yoon, B. J. Suh, *Polyhedron* **2004**, *23*, 1333–1339.

[2] S. Lorenz, B. Plietker, *ChemCatChem* **2016**, *8*, 3203–3206.

[3] J. Yadav, D. J. Mondal, S. Konar, *Chem. Commun.* **2021**, *57*, 5925–5928.

[4] O. Kahn, Molecular Magnetism. VCH-Verlag, Weinheim, New York **1993**.

[5] M. Nihei, M. Ui, N. Hoshino, H. Oshio, *Inorg. Chem.* **2008**, *47*, 6106–6108.

[6] J. I. Steinfeld, J. S. Francisco, W. L. Hase, *Chemical Kinetics and Dynamics*, Prentice Hall Upper Saddle River, NJ, **1999**.

[7] J. Franz, M. Oelschlegel, J. P. Zobel, S.-A. Hua, J.-H. Borter, L. Schmid, G. Morselli, O. S. Wenger, D. Schwarzer, F. Meyer, L. González, *J. Am. Chem. Soc.* **2024**, *146*, 11272–11288.

[8] Gaussian 16, Revision C.01, M. J. Frisch, G. W. Trucks, H. B. Schlegel, G. E. Scuseria, M. A. Robb, J. R. Cheeseman, G. Scalmani, V. Barone, G. A. Petersson, H. Nakatsuji, X. Li, M. Caricato, A. V. Marenich, J. Bloino, B. G. Janesko, R. Gomperts, B. Mennucci, H. P. Hratchian, J. V. Ortiz, A. F. Izmaylov, J. L. Sonnenberg, D. Williams-Young, F. Ding, F. Lipparini, F. Egidi, J. Goings, B. Peng, A. Petrone, T. Henderson, D. Ranasinghe, V. G. Zakrzewski, J. Gao, N. Rega, G. Zheng, W. Liang, M. Hada, M. Ehara, K. Toyota, R. Fukuda, J. Hasegawa, M. Ishida, T. Nakajima, Y. Honda, O. Kitao, H. Nakai, T. Vreven, K. Throssell, J. A. Montgomery Jr., J. E. Peralta, F. Ogliaro, M. J. Bearpark, J. J. Heyd, E. N. Brothers, K. N. Kudin, V. N. Staroverov, T. A. Keith, R. Kobayashi, J. Normand, K. Raghavachari, A. P. Rendell, J. C. Burant, S. S. Iyengar, J. Tomasi, M. Cossi, J. M. Millam, M. Klene, C. Adamo, R. Cammi, J. W. Ochterski, R. L. Martin, K. Morokuma, O. Farkas, J. B. Foresman, D. J. Fox, Gaussian, Inc., Wallingford CT, **2016**.

[9] R. Li, J. Zheng, D. G. Truhlar, *Phys. Chem. Chem. Phys.* **2010**, *12*, 12697–12701.

[10] F. Weigend, R. Ahlrichs, *Phys. Chem. Chem. Phys.* **2005**, *7*, 3297–3305.

[11] P. Salvador, E. Ramos-Cordoba, M. Montilla, L. Pujal, M. Gimferrer, *J. Chem. Phys.* **2024**, *160*, 172502.

[12] P. Salvador, E. Ramos-Cordoba, *J. Chem. Phys.* **2013**, *139*, 071103.

[13] N.S. Hush, *Prog. Inorg. Chem.* **1967**, *8*, 391–444.

[14] B. S. Brunschwig, N. Sutin, *Coord. Chem. Rev.* **1999**, *187*, 233–254.
