# Supplementary figures and images for: Cooperativity of Electron Transfer Coupled Spin Transitions in a Tetranuclear Fe/Co Prussian Blue Analogue Revealed by Ultrafast Spectroscopy
Source: Angew Chem Int Ed Engl. 2025 May 9;64(27):e202505813. doi: 10.1002/anie.202505813 (PMC12207354; doi:10.1002/anie.202505813)

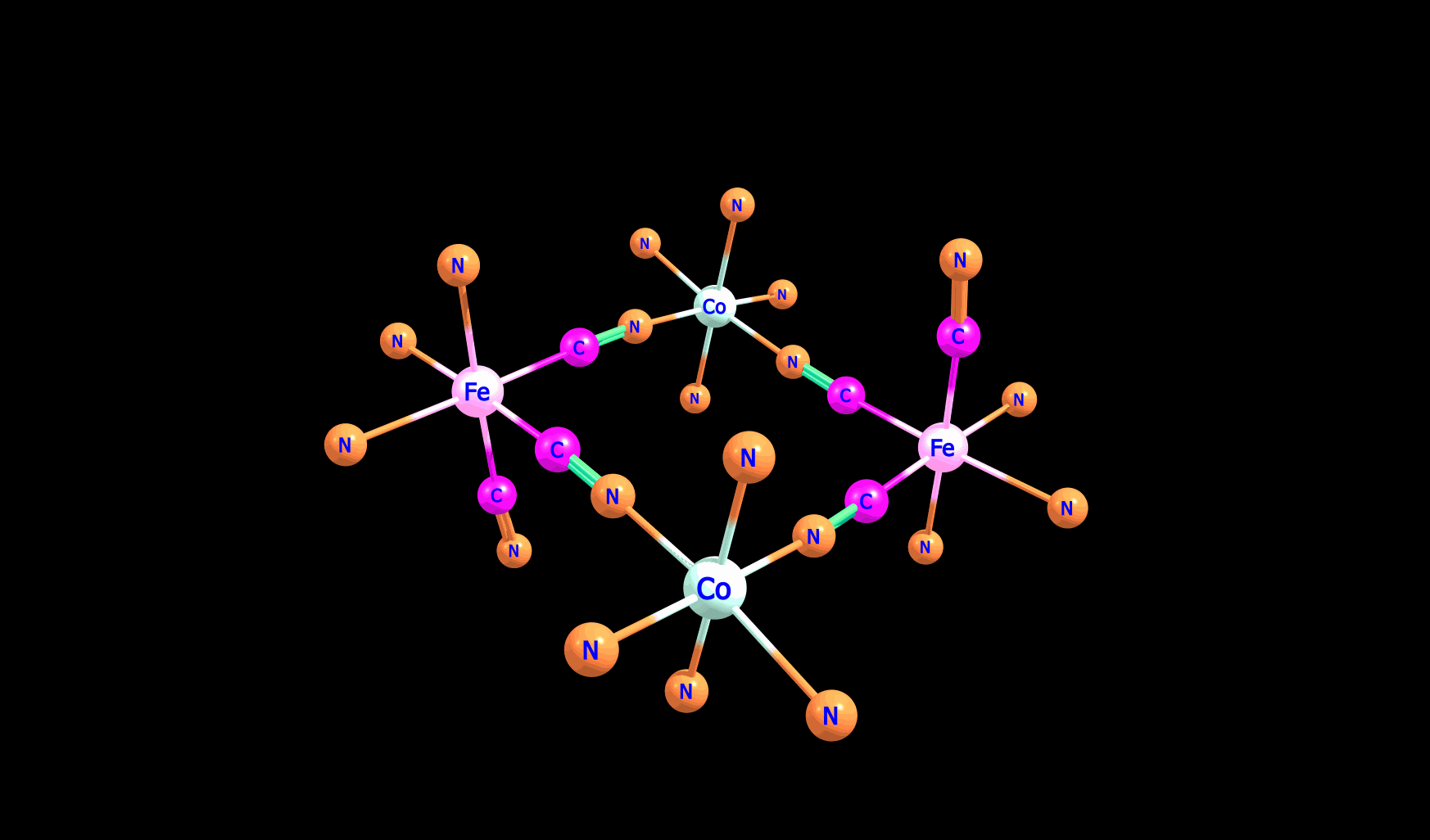

Supplement: Supplementary file 2 — Supporting Information [file ANIE-64-e202505813-s002.gif]
